# Novel visualized quantitative epigenetic imprinted gene biomarkers diagnose the malignancy of ten cancer types

**DOI:** 10.1186/s13148-020-00861-1

**Published:** 2020-05-24

**Authors:** Rulong Shen, Tong Cheng, Chuanliang Xu, Rex C. Yung, Jiandong Bao, Xing Li, Hongyu Yu, Shaohua Lu, Huixiong Xu, Hongxun Wu, Jian Zhou, Wenbo Bu, Xiaonan Wang, Han Si, Panying Shi, Pengcheng Zhao, Yun Liu, Yongjie Deng, Yun Zhu, Shuxiong Zeng, John P. Pineda, Chunlin Lin, Ning Zhou, Chunxue Bai

**Affiliations:** 1 Department of Pathology, Ohio State University Wexner Medical Center, Columbus, OH 43210 USA; 2 Epigenetics Lab, Chinese Alliance Against Lung Cancer, 6th Floor, Building 5, No.66, Jinghuidongdao Road, Wuxi, 214135 Jiangsu China; 3 Department of Urology, Changhai Hospital, Navy Medical University, Shanghai, 200433 China; 4 Department of Oncology, Johns Hopkins University School of Medicine, Baltimore, MD 21207 USA; 5 Departments of Endocrinology, Ultrasound and Pathology, JiangYuan Hospital Affiliated to Jiangsu Institute of Nuclear Medicine, Wuxi, 214063 Jiangsu China; 6 Department of Pathology, Changzheng Hospital, Navy Medical University, Shanghai, 200003 China; 7 Department of Pulmonary Medicine, Shanghai Respiratory Research Institute, Zhongshan Hospital, Fudan University, 180 Fenglin Road, Shanghai, 200032 China; 8 Department of Medical Ultrasound, Shanghai Tenth People’s Hospital, Ultrasound Research and Education Institute, Tongji University School of Medicine, Shanghai, 200072 China; 9 Hospital for Skin Disease, Institute of Dermatology, Chinese Academy of Medical Science, Peking Union Medical College, Nanjing, 210042 China; 10 Department of Molecular Medicine, The University of Texas Health Science Center at San Antonio, San Antonio, TX 78245 USA

**Keywords:** Epigenetics, Genomic imprinting, Biallelic expression, Multiallelic expression, Cancer biomarker

## Abstract

**Background:**

Epigenetic alterations are involved in most cancers, but its application in cancer diagnosis is still limited. More practical and intuitive methods to detect the aberrant expressions from clinical samples using highly sensitive biomarkers are needed. In this study, we developed a novel approach in identifying, visualizing, and quantifying the biallelic and multiallelic expressions of an imprinted gene panel associated with cancer status. We evaluated the normal and aberrant expressions measured using the imprinted gene panel to formulate diagnostic models which could accurately distinguish the imprinting differences of normal and benign cases from cancerous tissues for each of the ten cancer types.

**Results:**

The Quantitative Chromogenic Imprinted Gene In Situ Hybridization (QCIGISH) method developed from a 1013-case study which provides a visual and quantitative analysis of non-coding RNA allelic expressions identified the guanine nucleotide-binding protein, alpha-stimulating complex locus (*GNAS*), growth factor receptor-bound protein (*GRB10*), and small nuclear ribonucleoprotein polypeptide N (*SNRPN*) out of five tested imprinted genes as efficient epigenetic biomarkers for the early-stage detection of ten cancer types. A binary algorithm developed for cancer diagnosis showed that elevated biallelic expression (BAE), multiallelic expression (MAE), and total expression (TE) measurements for the imprinted gene panel were associated with cell carcinogenesis, with the formulated diagnostic models achieving consistently high sensitivities (91–98%) and specificities (86–98%) across the different cancer types.

**Conclusions:**

The QCIGISH method provides an innovative way to visually assess and quantitatively analyze individual cells for cancer potential extending from hyperplasia and dysplasia until carcinoma in situ and invasion, which effectively supplements standard clinical cytologic and histopathologic diagnosis for early cancer detection. In addition, the diagnostic models developed from the BAE, MAE, and TE measurements of the imprinted gene panel *GNAS*, *GRB10*, and *SNRPN* could provide important predictive information which are useful in early-stage cancer detection and personalized cancer management.

## Background

Globally, cancer is one of the leading causes of death, with worldwide incidence rate increasing rapidly. Early detection and diagnosis can curb cancer mortality and increase chances for curative treatment. The development of molecular biology has provided a better way to diagnose cancer earlier than traditional histopathology [1–3]. Clinical application of gene markers such as mutations in *TP53*, *EGFR*, *KRAS*, *BRCA*, and other genes has greatly improved cancer diagnosis, prognostication, and prediction of therapeutic responses to specific treatments [4–7]. Whole genome or exome sequencing has been added to the diagnostic portfolio for classifying tumors [8]. There is also a strong evidence that epigenetic changes, such as DNA methylation or histone methylation and acetylation, often occur at the precancerous stages and promote carcinogenesis which may be responsible for up to 80% of cancers [9].

Genomic imprinting is one type of epigenetic regulator that plays important roles in mammalian embryo development. In normal post embryonic somatic cells, imprinted genes are expressed either from the maternal or paternal allele only, because one copy is silenced through epigenetic markers such as DNA methylation and histone acetylation [10]. In diseased states, the normally silenced copies of some imprinted genes may be aberrantly activated via demethylation, leading to expression from both alleles. This phenomenon is normally named loss of imprinting (LOI) and has been reported to occur in various human cancers [11]. For example, the *H19/*insulin-like growth factor 2 (*IGF2*) and insulin-like growth factor 2 receptor (*IGF2R*) imprinted gene network is involved in colorectal, prostate, lung and breast cancers [12–14]. There is evidence suggesting that LOI of the guanine nucleotide-binding protein, alpha-stimulating complex locus (*GNAS*) gene may increase the risk of thyroid cancer, osteosarcoma, skin cancer, and neurofibromatosis [15]. Aberrant methylation and increased LOI of growth factor receptor-bound protein 10 (*GRB10*) are related to breast cancer [16]. In addition, hypomethylation of small nuclear ribonucleoprotein polypeptide N (*SNRPN*) gene in germ cell tumors was found to result in LOI [17].

Despite the growing evidence supporting a causal role of LOI in tumorigenesis, imprinted genes have not been used as diagnostic or predictive cancer biomarkers. Current methods, including bisulfite DNA sequencing and RNA sequencing [18, 19] have potential clinical value as they can provide indirect indicators such as methylation status or maternal/paternal expression ratios. However, an easy, intuitive, and quantitative method to detect the aberrant expression of imprinted genes in clinical samples is needed for clinical application.

An in situ hybridization (ISH)-based technique targeting the non-coding intronic nascent RNAs to visualize the transcription loci of imprinted genes in cell nuclei was developed to analyze the allele-specific expression of imprinted genes and the behavior of X chromosome [20]. Based on this technology, we designed a novel quantification approach to identify the presence of allelic expressions through the transcription signals in the nuclei. Using this method, most cells of normal tissues would show single or no allelic expression. However, elevated allelic expressions would be observed for cells from non-normal tissues. Based on these results, we developed the biallelic (BAE), multiallelic (MAE), and total (TE) expression scores and evaluated their relationship with cancer. We initially screened a panel of imprinted genes composed of *GNAS*, *GRB10*, *SNRPN*, *IGF2*, and *IGF2R* using our Quantitative Chromogenic Imprinted Gene In Situ Hybridization (QCIGISH) method by evaluating the aberrant expression of imprinting loci in a variety of cancer tissue samples, and distinguishing the imprinting differences of normal and benign cases from cancerous tissues. This novel technique could provide a quantitative determination of cancer status and serve to complement traditional cytology and histopathology examinations.

## Results

### Patient cohort and sample collection

**Fig. 1:**
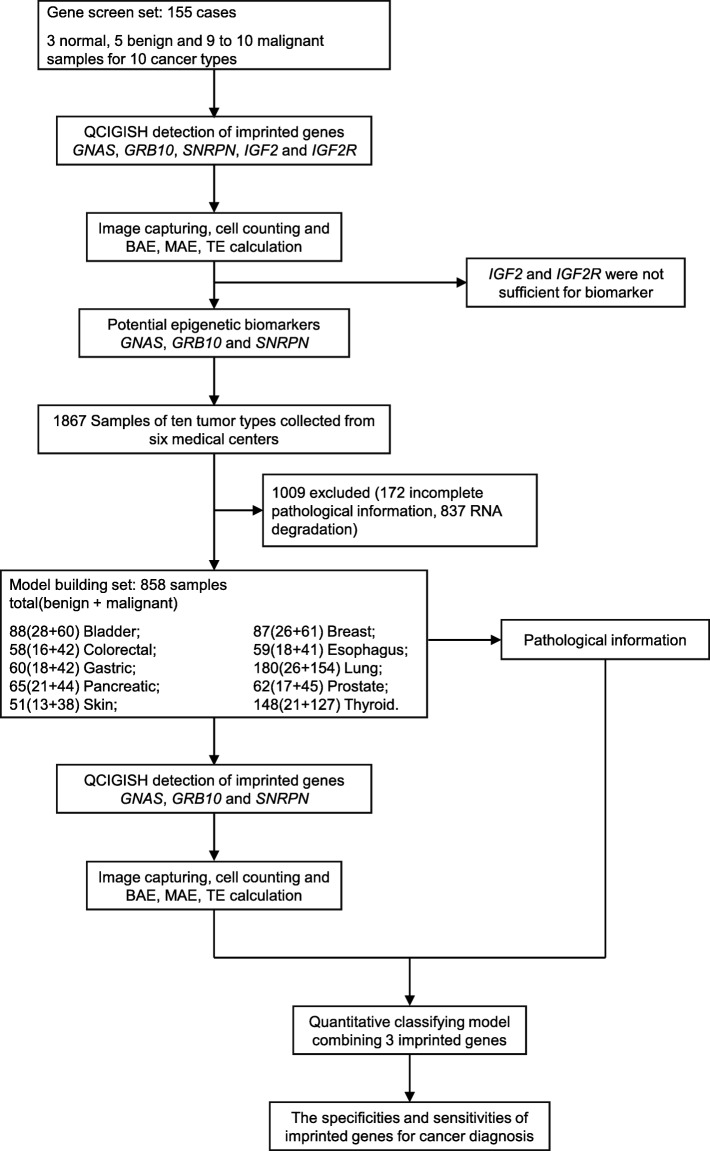
Study design for imprinted gene biomarker screening and diagnostic model development

In this study, 1013 patients with biopsies of known normal and benign (260 cases) and cancerous (753 cases) diagnosis from ten different solid cancer types (bladder, breast, colorectal, esophageal, gastric, lung cancer, pancreatic, prostate, skin, and thyroid cancers) were recruited from six participating hospitals (Fig. 1 and Table 1) under the direction of the institutional principal investigator (Dr. Bai). All methods were carried out in accordance with relevant guidelines and regulations. All experimental protocols of this retrospective study were approved by each hospital or institution’s licensing ethics committee review boards (Shanghai Changhai Hospital Ethics Committee, Shanghai Changzheng Hospital Ethics Committee, Zhongshan Hospital Fudan University Ethics Committee, Institute of Dermatology, Chinese Academy of Medical Sciences Ethics Committee, Jiangsu Institute of Nuclear Medicine Ethics Committee, Shanghai Tenth People’s Hospital Ethics Committee; approval number in Table 1). All subjects were above 18 and informed consent was obtained from all subjects. Fine-needle aspiration samples, core-needle aspiration samples, and conventional biopsies of ten specific tissue types (bladder, breast, colorectal, esophageal, gastric, lung, pancreatic, prostate, skin, and thyroid) were analyzed. Surgical samples were processed by standard formalin-fixed paraffin-embedded (FFPE) techniques. For the study of bladder cancer, additional urine samples were collected from cancer patients and from a cohort of healthy volunteers. With the exception of skin samples, all pathology diagnoses by local expert pathologists, were independently reviewed and confirmed by three reference pathologists (Drs. Shen, Yu, and Lu) according to the WHO criteria. The pathologic diagnosis of skin samples was provided by the Institute of Dermatology, Chinese Academy of Medical Sciences.

**Table 1:** Sources of tumor cases for cancer-specific diagnostic model building in this study

Tissue type	Case numbers		Hospitals	IRB/IEC number
	Benign*	Malignant		
Bladder	31	60	Shanghai Changhai Hospital	CHEC2019-029
Breast	26	61	Shanghai Changzhen Hospital	2018SL015
Colorectal	16	42	Shanghai Changzhen Hospital	2018SL015
Esophagus	18	41	Shanghai Changzhen Hospital	2018SL015
Gastric	18	42	Shanghai Changzhen Hospital	2018SL015
Lung	26	154	Shanghai Changzhen Hospital; Shanghai Institute of Respiratory Diseases, Shanghai Zhongshan Hospital	2018SL015, 2017-035R
Pancreatic	21	44	Shanghai Changzhen Hospital	2018SL015
Prostate	17	45	Shanghai Changzhen Hospital	2018SL015
Skin	13	38	Institute of Dermatology, Chinese Academy of Medical Sciences	2018-LKS-014
Thyroid	24	127	Jiangsu Jiangyuan Hospital, Shanghai Tenth People’s Hospital	YL201811, SHSY-IEC-4.1/19-6/01
Totals	204	654		

*Benign samples were non-cancerous tissues from patients with benign lesions and used as negative control. Normal samples for gene screening were obtained adjacent to the benign lesions

### Differential allelic expression of imprinted genes observed in normal, benign, and malignant samples

**Fig. 2:**
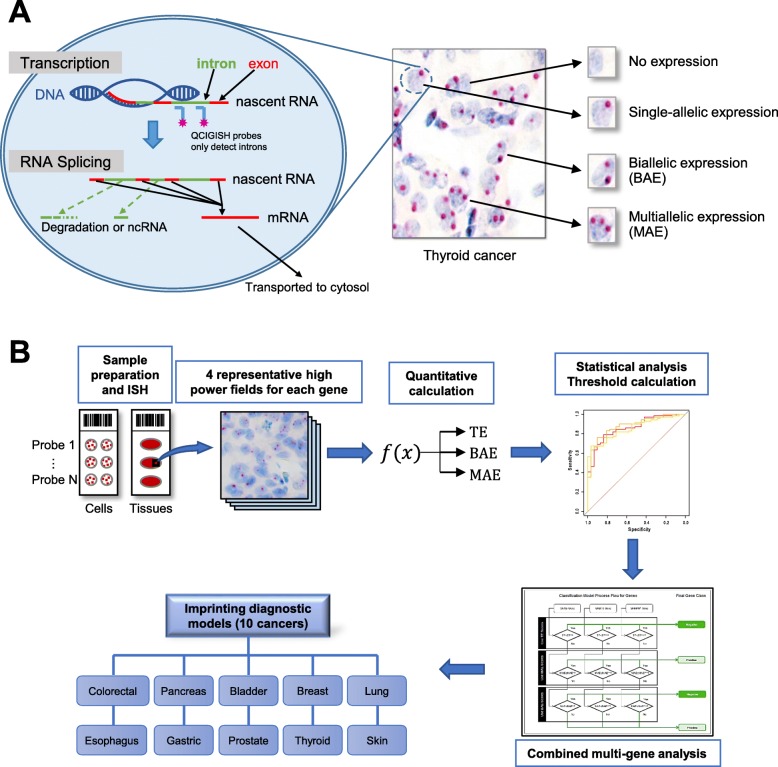
QCIGISH principle and workflow. **a** Different imprinted gene expression status and ISH visualized signals in thyroid cancer cells. **b** Workflow of imprinting detection and diagnostic model building

**Fig. 3:**
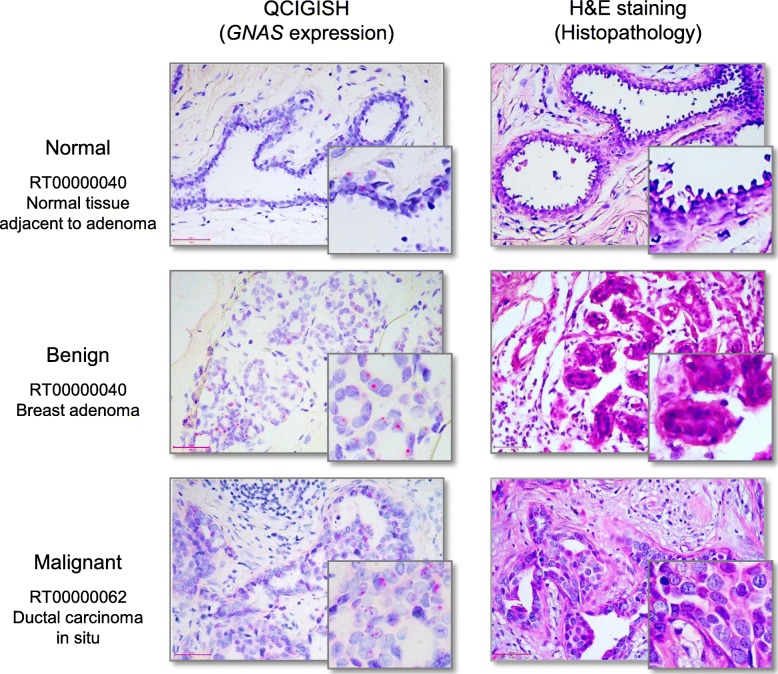
A comparative example of the imprinted gene expression and histopathology for normal, benign, and malignant cases illustrated using breast tissue samples. The left panels showed the allelic expression status of imprinted gene *GNAS*, and the right panels showed the corresponding standard hematoxylin-eosin (H&E) staining morphology

**Fig. 4:**
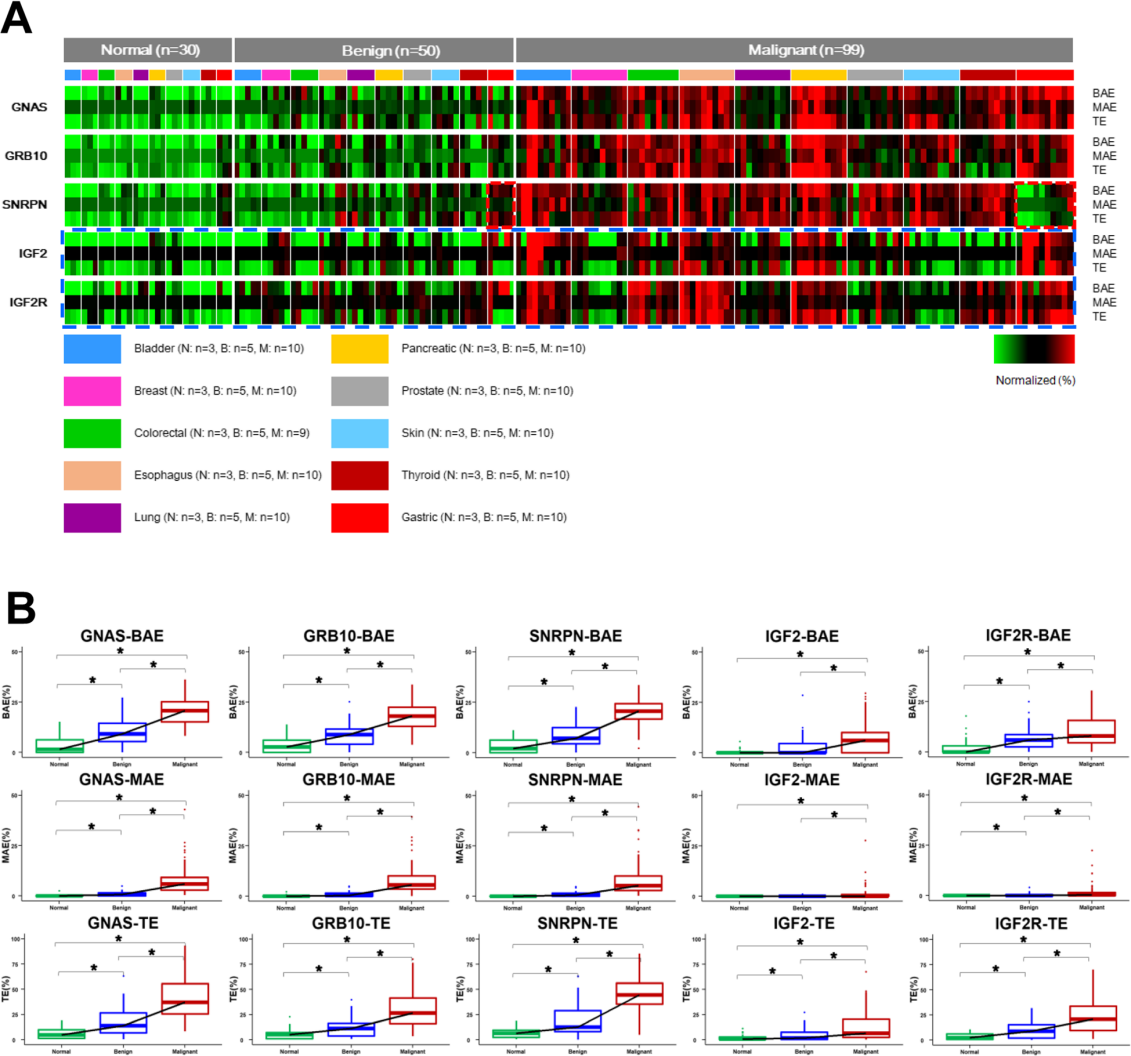
Comparison of the expression status of imprinted genes *GNAS*, *GRB10*, *SNRPN*, *IGF2*, and *IGF2R* in the gene screening set. **a** Heat map showing the expression status of imprinted gene *GNAS*, *GRB10*, *SNRPN*, *IGF2*, and *IGF2R*. N, normal samples; B, benign samples; M, malignant samples. Gastric cancers and benign controls are framed with red dashed lines. Additional imprinted genes *IGF2* and *IGF2R* studied are framed with blue dashed lines. **b** Box plot showing the expression status of imprinted genes in normal, benign, and malignant samples. *p < 0.01

To analyze the allelic expression of imprinted genes in cancer, we selected 5 imprinted genes *GNAS*, *GRB10*, *SNRPN*, *IGF2*, and *IGF2R* which were mostly reported to be associated with cancer [12–17]. The expressions of these 5 imprinted genes were evaluated on cancers of ten solid tumor types: bladder, breast, colorectal, esophagus, gastric, lung, pancreatic, prostate, skin, and thyroid (Fig. 2). For each tumor type, 9 to 10 cancer samples, 5 benign, and 3 pathologically identified normal controls which were obtained near the benign lesions were tested (Fig. 1). The differences in gene expression ISH staining patterns between normal, benign, and malignant samples were visually observed (sample images provided in Fig. 3), and were additive to the standard histologic changes by H&E staining. In normal samples, the *GNAS*, *GRB10*, *SNRPN*, *IGF2*, and *IGF2R* imprinted genes showed mostly no signal including a considerable number of cells with single signal (single allelic expression, SAE) and only a few cells expressing two signals (biallelic expression, BAE). Increased SAE and BAE with a few cells showing three or more signals (multiallelic expression, MAE) were however observed for benign cases. In contrast, BAE and MAE were shown to have significantly increased for malignant samples (Fig. 3 and Fig. S2–S4). Statistical analysis showed a significant increase in BAE, MAE, and total expression (TE) scores between normal and benign (*p* < 0.01, Table S1 and Fig. 4), and benign and malignant cases (*p* < 0.01, Table S1 and Fig. 4), respectively. When comparing normal samples with benign lesions, the computed *p* values for TE and BAE were comparatively lower than MAE (Table S1). The observed increase in TE and BAE scores are more efficient biomarkers for benign lesions as compared to MAE. Very low *p* values (<2.2e− 16) were also consistently obtained for BAE, MAE, and TE when comparing benign and malignant cases (Table S1) signifying their efficiency in detecting cancer. Statistical test results also showed significantly higher expressions for the *GNAS*, *GRB10*, *SNRPN*, *IGF2*, and *IGF2R* genes in malignant cases when compared with normal and benign samples (*p* < 0.01, Table S1). The computed *p* values for *GNAS*, *GRB10*, and *SNRPN* were however noted to be comparatively lower than those obtained for the *IGF2* and *IGF2R* genes (Table S1). One particular exception is for the *SNRPN* gene expression in gastric cancer. We observed a decrease of the BAE, MAE, and TE scores of *SNRPN* in advanced gastric cancer which resulted in the exclusion of this particular set of observations in this evaluation (Table S1 and Fig. 4). This finding may potentially suggest a different behavior of *SNRPN* in gastric cancer which needs further investigation.

### Imprinted genes *GNAS*, *GRB10*, and *SNRPN* identified as efficient cancer biomarkers for clinical application

In order to find useful and efficient cancer biomarkers for clinical applications, we generated the ROC curves for the *GNAS*, *GRB10*, *SNRPN*, *IGF2*, and *IGF2R* imprinted genes using the individual BAE, MAE, and TE measurements for all the different cancer types combined (Fig. S5). Based on the optimal thresholds provided by the ROC curves for the BAE, MAE, and TE measurements (Table S2), we developed negative/positive classification models for each of the *GNAS*, *GRB10*, *SNRPN*, *IGF2*, and *IGF2R* imprinted genes. In some cases, when TE is relatively low (lower than 5% as noted in certain cases), we observed a few cells with two or three signals which do not directly link to malignancy. In effect, the BAE and MAE measurements tend to increase, as computed based from the given equations, which could result to false positives. So, a TE threshold was selected as a first restriction in classifying benign from malignant cases to address such issue. Samples with TE scores lower than the defined threshold values for each gene were classified under the negative class (Fig. 6). For the remaining samples, if either the BAE or the MAE scores were above the threshold values, these were identified under the positive class. When both the BAE and MAE scores are below threshold, samples were considered negative (Fig. 6). To evaluate the effectiveness of the diagnostic models developed from each individual gene, we considered sensitivities and specificities higher than 80% to be useful for the study. After evaluating and adjusting the threshold values to achieve a specificity of at least 80% for each gene, sensitivity values of > 80% were obtained for *GNAS*, *GRB10*, and *SNRPN*, while < 60% sensitivities were computed for *IGF2* and *IGF2R* (Table S3). Using these results, imprinted genes *GNAS*, *GRB10*, and *SNRPN* were identified as the more efficient cancer biomarkers over *IGF2* and *IGF2R*, specifically using our QCIGISH method.

### Quantitative imprinting diagnostic models built using imprinted genes *GNAS*, *GRB10*, and *SNRPN*

**Fig. 5:**
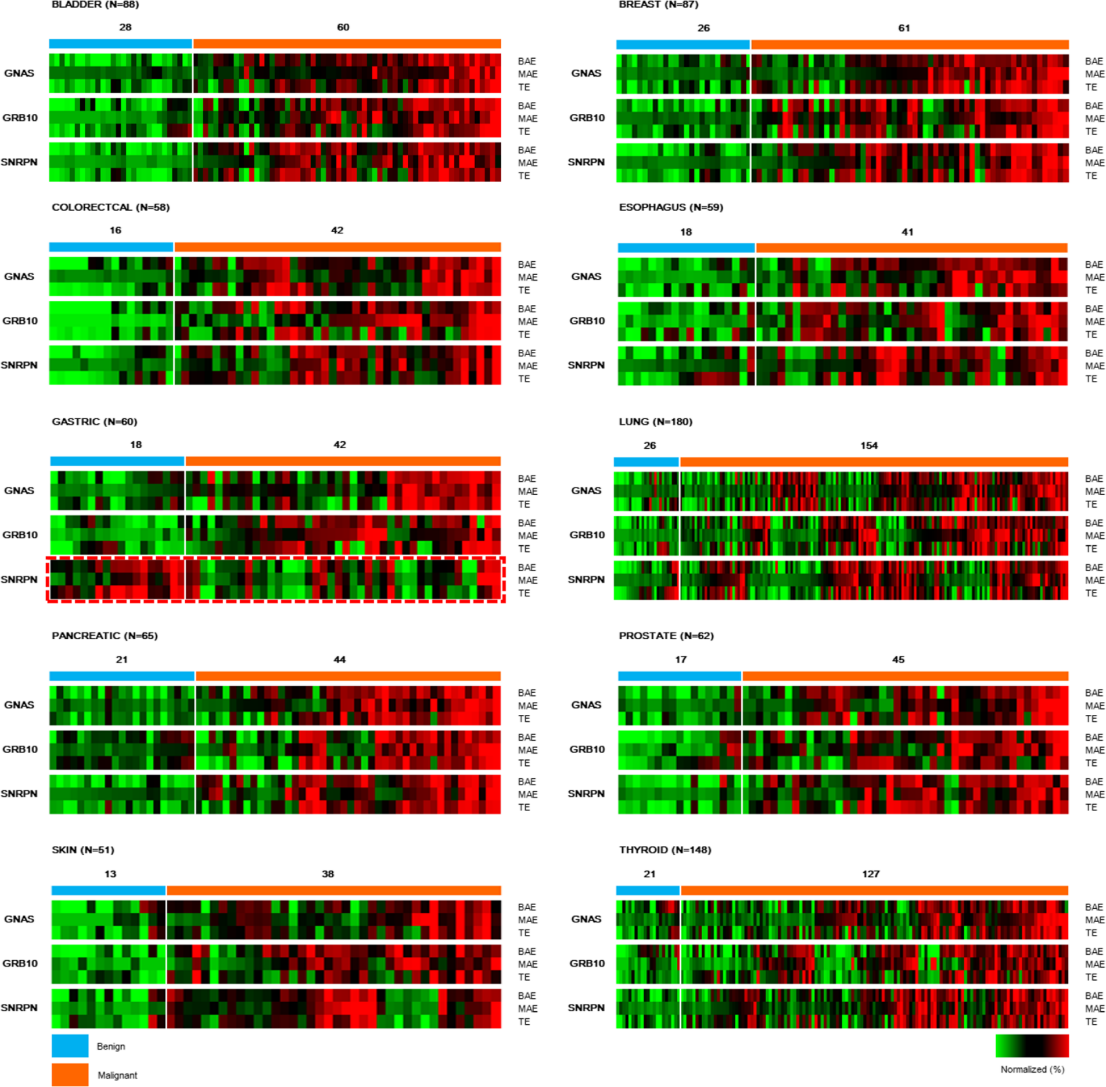
Comparison of the expression status of imprinted genes *GNAS*, *GRB10*, and *SNRPN* in the diagnostic model building set. Benign cases were indicated by blue bars, and malignant cases were indicated by orange bars. Gastric cancers and their benign controls are framed with red dashed lines

We further analyzed the expression of imprinted genes *GNAS*, *GRB10*, and *SNRPN* in 204 benign and 654 malignant samples across ten cancer types. The BAE, MAE, and TE measurements for each gene were individually normalized as *z* scores across all samples for each cancer type and were shown in heat maps (Fig. 5). For each cancer type, the benign and malignant cases were separately labeled with each heat map column corresponding to the same observation. The observations were arranged by clustering together those with similar characteristics in terms of their *GNAS*, *GRB10*, and *SNRPN* expressions. Statistical analysis showed that the TE, BAE, and MAE scores of imprinted genes *GNAS*, *GRB10*, and *SNRPN* were significantly increased in malignant samples for almost all ten cancer types (*p* < 0.01), with the only exception of *SNRPN* in gastric cancer (Table S4). These results confirmed that the biallelic and multiallelic expressions of *GNAS*, *GRB10*, and *SNRPN* could be effectively used as cancer biomarkers for distinguishing benign from malignant tumors.

**Fig. 6:**
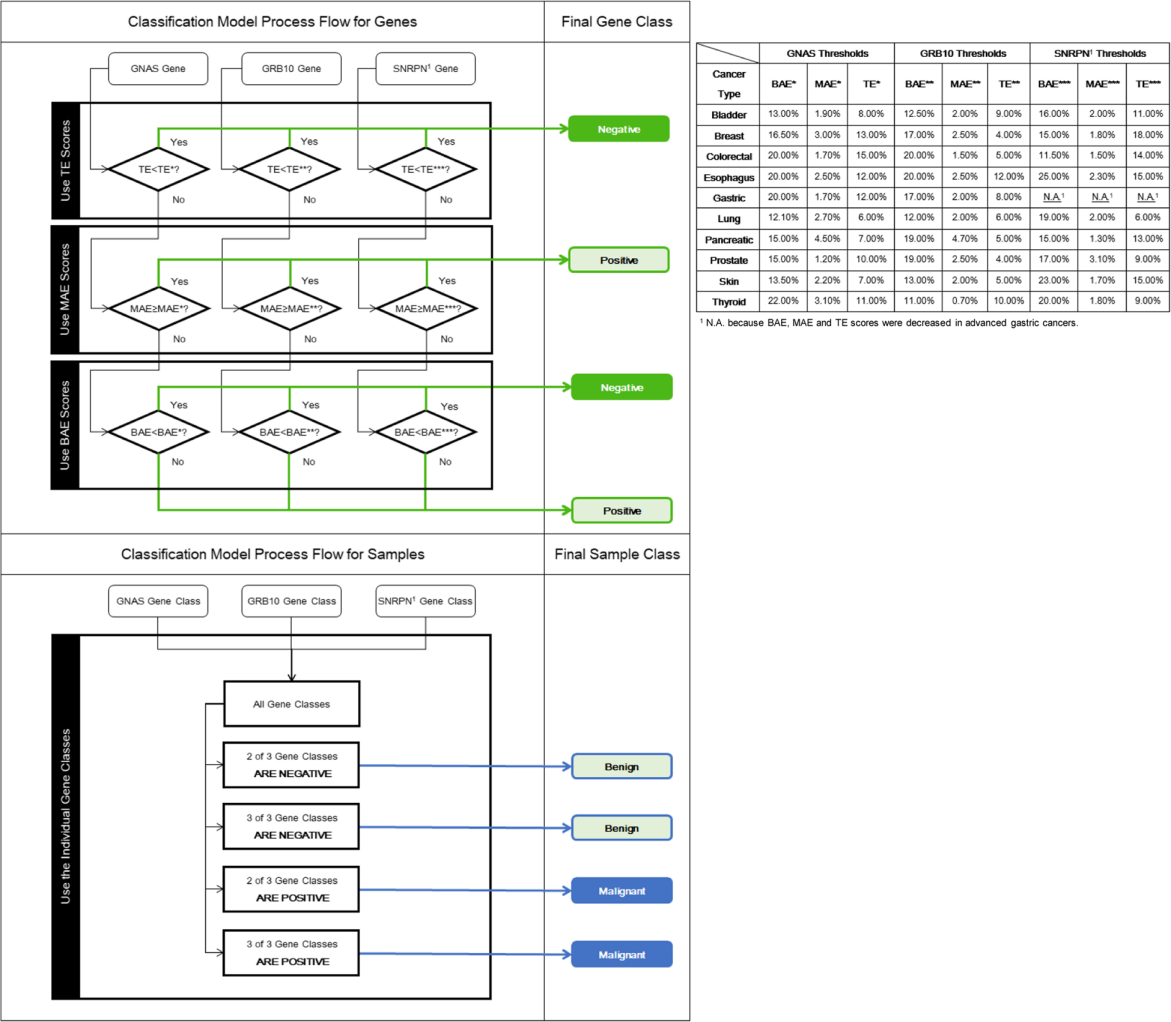
Cancer diagnostic model using the imprinted genes *GNAS*, *GRB10*, and *SNRPN* for the ten cancer types

To build the diagnostic models for the different cancer types, we refined the thresholds of the primary classification models according to the ROC curves for different cancers (Fig. S6). Although the BAE, MAE, and TE scores showed a significant difference between benign and malignant samples, overlaps were observed. Considering the potential differences of the gene expressions among cancer types, we refined the thresholds for each individual gene with emphasis on sensitivity. With the optimal thresholds initially computed for the BAE, MAE, and TE measurements for each gene per cancer type (Table S5–S14), we evaluated and adjusted the individual thresholds to generally achieve > 70% sensitivity and specificity. Only those cases with two genes or more which exhibited a positive class are classified as malignant (Fig. 6 and Table S15–S16). However, as the BAE, MAE, and TE scores of the *SNRPN* gene in advanced gastric cancer were previously noted to have behaved in opposite of those observed from other cancer types, this gene was specifically not included in the classification model for gastric cancer, and only those with both *GNAS*- and *GRB10*-positive classes can be identified as malignant (Fig. 6). Based from the results, we have noted low specificities for certain genes and cancer types including *GRB10* gene for lung cancer and *GNAS* gene for thyroid cancers. However, upon evaluation, the specificities could be effectively improved when these particular genes were combined with the other genes together in the diagnostic model. We are still pursuing further studies involving new epigenetic biomarkers to improve cancer diagnostic specificities.

### Imprinting diagnostic models exhibit high sensitivity and specificity in cancer diagnosis

After building the imprinting diagnostic models based on our dataset, we measured the sensitivities and specificities in the model building sets (Table 2 and Table S16). The models exhibited sensitivities higher than 90% in all the ten cancers, along with very high specificities, being > 90% in six of ten cancer types and ≥ 85% in four cancers (esophagus, lung, prostate, and thyroid). The overall sensitivity of the ten cancer types was 94% and overall specificity was 92%, with a diagnostic accuracy of 93%. These models will be further validated and refined in later cancer-specific studies with larger sample sets.

**Table 2:** Sensitivities and specificities of QCIGISH diagnostic models in different tumors

Type of tumor	Sensitivity	Specificity	Benign		Malignant	
			Imprinting negative	Imprinting positive	Imprinting negative	Imprinting positive
Bladder	98%	96%	27	1	1	59
Breast	98%	96%	25	1	1	60
Colorectal	98%	94%	15	1	1	41
Esophagus	95%	89%	16	2	2	39
Gastric	93%	94%	17	1	3	39
Lung	92%	88%	23	3	13	141
Pancreatic	93%	90%	19	2	3	41
Prostate	93%	88%	15	2	3	42
Skin	97%	92%	12	1	1	37
Thyroid	91%	86%	18	3	12	115
Total	94%	92%	187	17	40	614

## Discussion

Current epigenetics research focuses on DNA methylation, histone acetylation, chromosomal architecture, miRNAs, and lncRNAs [21–23]. Many technologies have been developed to analyze the relationship between epigenetic changes and gene expression levels. The epigenetic changes detected by these technologies can infer the altered expression status of genes; some of the changes, such as DNA methylation and miRNA, are currently being tested in clinical practice [24, 25]. However, there is still a lack of clear cancer biomarkers to meet the requirements for precision diagnostic medicine. In this study, we applied a QCIGISH method targeting intronic non-coding RNA to visualize and quantify the number of allelic expressions of imprinted genes related to carcinogenesis. The diagnostic models built on the BAE, MAE, and TE scores of imprinted genes showed consistently high accuracy across ten cancer types, therefore suggesting QCIGISH as an effective and readily suitable tool for clinical applications.

Our preliminary results from 753 cases of ten cancer types showed that biallelic and multiallelic expressions of imprinted genes were dramatically increased in malignant samples as compared to 260 organ-specific benign and normal counterparts. This interesting finding validates the difference in signal information being provided between non-coding RNA from introns and coding RNA from exons. The involvement of introns in alternatively spliced mRNAs has been reported in several cancers and can be linked to epigenetics [26]. Our results provided additional evidence that expression of introns could be used as clear epigenetic markers for cancer. However, the underlying mechanisms are not yet clear and need further study.

From the analysis of the proportion of cells with different expression status, we noted that the observed increase in TE and BAE scores are more efficient biomarkers for benign lesions as compared to MAE. In addition, BAE, MAE, and TE are all elevated for carcinogenesis. The biallelic and multiallelic expressions of imprinted genes observed using the QCIGISH method may include regular LOI, but it could also involve the amplification of the normally activated allele with the other allele remaining silenced [27]. However, our results clearly showed that BAE and MAE scores are related to cancer malignancy.

For our study, all the *GNAS*, *GRB10*, and *SNRPN* genes shared a similar expression pattern in 753 cases of ten cancers. It is reasonable to believe that this phenomenon exists in other cancers as well, but more accurate and precise diagnostic models may require the incorporation of additional genes for different cancers types. *IGF2* and *IGF2R* are usually referred in the studies on imprinted genes and cancers [12–14], which we have also tested in our samples. Although *IGF2* and *IGF2R* genes were also observed to be related to cancer, they were not the best biomarkers specifically using our QCIGISH method. We therefore selected *GNAS*, *GRB10*, and *SNRPN* genes as the more efficient cancer biomarkers for our diagnostic model.

Based off of the results of this study, our preliminary diagnostic models will be upgraded to a more detailed grading scoring system defining different levels of malignancy, characterizing cell development from benign to early-stage cancer, and further validated in larger sample sets using presurgical cytology samples across different cancer types. More imprinted gene cancer biomarkers are also being tested and will be incorporated into the upgraded model. The present study conceptually opens a new diagnostic area in cancer and demonstrates strong potential for high-throughput clinical application. It is expected that this technology may go beyond a diagnostic technique to also provide information on prognostic and predictive markers of treatment response. We also anticipate the adoption of machine learning and artificial intelligence (AI) through the inclusion of automatic image recognition and big data analysis. The large-scale clinical application of imprinting diagnosis could complement current early cancer detection via serum biomarkers and imageological examination, which ultimately is one of the key steps in improving cancer prognosis and reducing cancer mortality.

## Conclusion

We presented a novel QCIGISH method in the current study that targets the non-coding intronic region of nascent RNAs to visualize and quantify the allelic expressions of imprinted genes in cancer development. For this investigation, we focused on the expression status of three imprinted genes—*GNAS*, *GRB10*, and *SNRPN* known to be associated with cancer status. The testing of the three imprinted gene panel on 1013 clinical samples, including 260 normal and benign, and 753 malignant cases from bladder, breast, colorectal, esophageal, gastric, lung, pancreatic, prostate, skin, and thyroid cancers demonstrated how the expression status of *GNAS*, *GRB10*, and *SNRPN* significantly changed in cancer cases versus their normal and benign tissue controls. We have specifically built cancer-specific diagnostic models based on the BAE, MAE, and TE scores, with the combination of these three genes yielding an overall sensitivity of 94% and specificity of 92%. While this initial study still needs confirmation in even larger cohorts of cancers and benign controls, we believe that our observations regarding biallelic and multiallelic expressions of imprinted cancer genes at the single-cell and regional density levels will become a practically useful and powerful clinical tool by effectively supplementing standard cytologic and histopathologic diagnosis for early-stage cancer detection. Based off of the research from this initial study, we have also begun to explore the incorporation of machine learning and artificial intelligence to efficiently automate our image analysis process. In addition, we have proceeded with a multi-center prospective presurgical clinical study specifically for lung and thyroid cancers involving a much larger cohort. With all these foregoing initiatives, we look forward to further establishing the suitability of our technology for future clinical applications.

## Methods

### Sample preparation for in situ hybridization (ISH)

Tissue blocks were prepared by standard FFPE sample preparation protocol following the RNAscope (Advanced Cell Diagnostics, ACD Bio, Newark, CA, USA) sample preparation procedures [28] and serially cut into 10-μm sections. The sections were then mounted on positively charged slides and dried overnight at room temperature (RT). The sections were deparaffinized in xylene and pretreated following the RNAscope sample preparation procedures.

Fine-needle aspiration samples from thyroid, breast, lung, pancreas and prostate, and bronchial brushing samples were fixed immediately after sampling in 10% NBF (neutral buffered formalin) for 48 h at RT. The samples were directly mounted onto positively charged slides and pretreated following the RNAscope sample preparation procedures [28].

Surgical biopsies from the thyroid, breast, and pancreas; endoscopic biopsies from the bladder, esophagus, stomach, colorectum, and lung; and core-needle aspirations from the breast were fixed immediately after sampling in 10% NBF for 48 h at RT. Cells dissociated from these tissues were mounted on positively charged slides and pretreated following the RNAscope sample preparation procedures [28].

Exfoliated uroepithelial cell samples from healthy volunteers and cancer patients were collected using the following protocol. After the first morning void, patients consumed 500 ml of water; then, 80 ml of the next urine was collected and fixed for 48 h at RT. The samples were then centrifuged at 8000×*g* for 15 min to pellet the cells. The cells were mounted on positively charged slides and pretreated following the RNAscope sample preparation procedures [28].

### In situ hybridization

The ISH probes were designed to target the introns of nascent RNAs from *GNAS*, *GRB10*, *SNRPN*, *IGF2*, and *IGF2R* [29] and were synthesized by ACD Bio. A positive control probe, PPIB (targeting *Homo sapiens* Peptidylprolyl Isomerase B, a ubiquitously expressed gene), and negative control probe, dapB (targeting *Bacillus subtilis* strain SMY dihydrodipicolinate reductase), were purchased from ACD Bio. For each sample, the five imprinted gene probes were hybridized individually with serially cut sections from the same tissue block using RNAscope 2.5 HD assay kit (ACD Bio) according to the manufacturer’s instructions [28]. The probe design as well as the pretreatment and hybridization condition were optimized for detecting single-stranded RNA and not for double-stranded genomic DNA wrapped by histones. RNase treatment was also performed to verify that the signals came from RNA (Fig. S1). The positive and negative control probes were both applied onto every slide. After hybridization, the signals were amplified via six steps and detected using chromogenic reagents (Fast Red for red signals and DAB for brown signals, ACD Bio) [28]. Each gene expressing site appears as a distinct red or brown dot under common bright field microscope (Fig. 2a).

### Data collection

After the images were captured under × 400 microscope, the number of nuclei containing no signal (no expression = *N*_0_), one signal (single allelic expression = *N*_1_), two signals (biallelic expression = *N*_2_), and more than two signals (multiallelic expression = *N*_2plus_) were counted manually from four representative high power fields per gene/sample (Fig. 2b). The technologists performing the counting were blinded to the diagnosis of the samples. The BAE, MAE, and TE were then calculated according to the following equations (Fig. 2b):

$$ \mathrm{BAE}=\frac{N_2}{N_1+{N}_2+{N}_{2\mathrm{plus}}}\times 100\% $$

$$ \mathrm{MAE}=\frac{N_{2\mathrm{plus}}}{N_1+{N}_2+{N}_{2\mathrm{plus}}}\times 100\% $$

$$ \mathrm{TE}=\frac{N_1+{N}_2+{N}_{2\mathrm{plus}}}{N_0+{N}_1+{N}_2+{N}_{2\mathrm{plus}}}\times 100\% $$

### Statistical analysis

Statistical analysis was proceeded to investigate for significant differences between the BAE, MAE, and TE scores among the *GNAS*, *GRB10*, *SNRPN*, *IGF2*, and *IGF2R* imprinted genes in normal controls, benign lesions, and malignant case groups for both the gene screening and model building data sets involving the various cancer types. Continuous variables were presented as medians with interquartile ranges (IQR). To facilitate the comparison between the aforementioned groups, a one-tailed robust rank-order nonparametric test was applied because the study parameters used continuous measurements and involved various pairs of small-sized independent samples with differing variabilities. A computed *p* of less than 0.01 indicates a significantly higher BAE, MAE, or TE level for the independent groups being compared. The heat maps were generated using Multiple Experiment Viewer (MeV) software [30]. The receiver operating characteristic (ROC) curves were generated through R code package pROC [31]. Classification thresholds for the BAE, MAE, and TE scores of each gene were determined according to the ROC curves (Fig. 2b). The classification models for each cancer were generated by combining the classification results of the three genes (Fig. 2b). Both sensitivity and specificity values were calculated using standard formulas. The analysis was conducted using R software (version 3.5.0).

## Supplementary information

**Additional file 1.** Supplementary Data

## Data Availability

The datasets used and analyzed during the current study are available from Dr. Ning Zhou (e-mail: zhou.ning@lisenid.com) on request.

## Abbreviations

QCIGISH: Quantitative Chromogenic Imprinted Gene In Situ Hybridization
SAE: Single allelic expression
BAE: Biallelic expression
MAE: Multiallelic expression
TE: Total expression
LOI: Loss of imprinting
*GNAS*: Guanine nucleotide-binding protein, alpha-stimulating complex locus
*GRB10*: Growth factor receptor-bound protein 10
*SNRPN*: Small nuclear ribonucleoprotein polypeptide N
*IGF2*: Insulin-like growth factor 2
*IGF2R*: Insulin-like growth factor 2 receptor

## References

[1] Heitzer E, Haque IS, Roberts CES, Speicher MR (2019). Current and future perspectives of liquid biopsies in genomics-driven oncology. Nat Rev Genet.

[2] Liu R, Zhang T, Zhu G, Xing M (2018). Regulation of mutant TERT by BRAF V600E/MAP kinase pathway through FOS/GABP in human cancer. Nat Commun.

[3] Teixeira VH, Pipinikas CP, Pennycuick A, Lee-Six H, Chandrasekharan D, Beane J, Morris TJ, Karpathakis A, Feber A, Breeze CE (2019). Deciphering the genomic, epigenomic, and transcriptomic landscapes of pre-invasive lung cancer lesions. Nat Med.

[4] Joerger AC, Fersht AR (2016). The p53 pathway: origins, inactivation in cancer, and emerging therapeutic approaches. Annu Rev Biochem.

[5] Shigematsu H, Lin L, Takahashi T, Nomura M, Suzuki M, Wistuba II, Fong KM, Lee H, Toyooka S, Shimizu N (2005). Clinical and biological features associated with epidermal growth factor receptor gene mutations in lung cancers. J Natl Cancer Inst.

[6] Zhuang R, Li S, Li Q, Guo X, Shen F, Sun H, Liu T (2017). The prognostic value of KRAS mutation by cell-free DNA in cancer patients: a systematic review and meta-analysis. PLoS One.

[7] Vasen HF, Tesfay E, Boonstra H, Mourits MJ, Rutgers E, Verheyen R, Oosterwijk J, Beex L (2005). Early detection of breast and ovarian cancer in families with BRCA mutations. Eur J Cancer.

[8] Wakai T, Prasoon P, Hirose Y, Shimada Y, Ichikawa H, Nagahashi M (2018). Next-generation sequencing-based clinical sequencing: toward precision medicine in solid tumors. Int J Clin Oncol.

[9] Feinberg AP (2018). The key role of epigenetics in human disease prevention and mitigation. N Engl J Med.

[10] Barlow DP, Bartolomei MS (2014). Genomic imprinting in mammals. Cold Spring Harb Perspect Biol.

[11] Jelinic P, Shaw P (2007). Loss of imprinting and cancer. J Pathol.

[12] Ribarska T, Goering W, Droop J, Bastian KM, Ingenwerth M, Schulz WA (2014). Deregulation of an imprinted gene network in prostate cancer. Epigenetics.

[13] Matouk IJ, Halle D, Gilon M, Hochberg A (2015). The non-coding RNAs of the H19-IGF2 imprinted loci: a focus on biological roles and therapeutic potential in Lung Cancer. J Transl Med.

[14] Ito Y, Koessler T, Ibrahim AE, Rai S, Vowler SL, Abu-Amero S, Silva AL, Maia AT, Huddleston JE, Uribe-Lewis S (2008). Somatically acquired hypomethylation of IGF2 in breast and colorectal cancer. Hum Mol Genet.

[15] Murrell A (2006). Genomic imprinting and cancer: from primordial germ cells to somatic cells. ScientificWorldJournal.

[16] Barrow TM, Barault L, Ellsworth RE, Harris HR, Binder AM, Valente AL, Shriver CD, Michels KB (2015). Aberrant methylation of imprinted genes is associated with negative hormone receptor status in invasive breast cancer. Int J Cancer.

[17] Lee SH, Appleby V, Jeyapalan JN, Palmer RD, Nicholson JC, Sottile V, Gao E, Coleman N, Scotting PJ (2011). Variable methylation of the imprinted gene, SNRPN, supports a relationship between intracranial germ cell tumours and neural stem cells. J Neuro-Oncol.

[18] Feng L, Lou J (1894). DNA methylation analysis. Methods Mol Biol.

[19] Goovaerts T, Steyaert S, Vandenbussche CA, Galle J, Thas O, Van Criekinge W, De Meyer T (2018). A comprehensive overview of genomic imprinting in breast and its deregulation in cancer. Nat Commun.

[20] Braidotti G (2001). RNA-FISH to analyze allele-specific expression. Methods Mol Biol.

[21] Huang B, Jiang C, Zhang R (2014). Epigenetics: the language of the cell?. Epigenomics.

[22] Liz J, Esteller M (2016). lncRNAs and microRNAs with a role in cancer development. Biochim Biophys Acta.

[23] Schmitt AD, Hu M, Ren B (2016). Genome-wide mapping and analysis of chromosome architecture. Nat Rev Mol Cell Biol.

[24] Cree IA, Uttley L, Buckley Woods H, Kikuchi H, Reiman A, Harnan S, Whiteman BL, Philips ST, Messenger M, Cox A (2017). The evidence base for circulating tumour DNA blood-based biomarkers for the early detection of cancer: a systematic mapping review. BMC Cancer.

[25] Mensah M, Borzi C, Verri C, Suatoni P, Conte D, Pastorino U, Orazio F, Sozzi G, Boeri M. MicroRNA based liquid biopsy: the experience of the plasma miRNA signature classifier (MSC) for lung cancer screening. Journal of visualized experiments : JoVE. 2017;128. 10.3791/56326 PMC5755225 29155727

[26] Narayanan SP, Singh S, Shukla S (2017). A saga of cancer epigenetics: linking epigenetics to alternative splicing. The Biochemical journal.

[27] Martin-Trujillo A, Vidal E, Monteagudo-Sanchez A, Sanchez-Delgado M, Moran S, Hernandez Mora JR, Heyn H, Guitart M, Esteller M, Monk D (2017). Copy number rather than epigenetic alterations are the major dictator of imprinted methylation in tumors. Nat Commun.

[28] Wang F, Flanagan J, Su N, Wang LC, Bui S, Nielson A, Wu X, Vo HT, Ma XJ, Luo Y (2012). RNAscope: a novel in situ RNA analysis platform for formalin-fixed, paraffin-embedded tissues. The Journal of molecular diagnostics : JMD.

[29] Soler M, Boque-Sastre R, Guil S (2017). RNA-FISH to study regulatory RNA at the site of transcription. Methods Mol Biol.

[30] Howe EA, Sinha R, Schlauch D, Quackenbush J (2011). RNA-Seq analysis in MeV. Bioinformatics.

[31] Chan BKC (2018). Applied statistics for human genetics using R. Adv Exp Med Biol.

