# Supplementary material for: Novel visualized quantitative epigenetic imprinted gene biomarkers diagnose the malignancy of ten cancer types
Source: Clin Epigenetics. 2020 May 24;12:71. doi: 10.1186/s13148-020-00861-1 (PMC7245932; doi:10.1186/s13148-020-00861-1)

Fig. S1. Quality control verification process for the QCIGISH method.

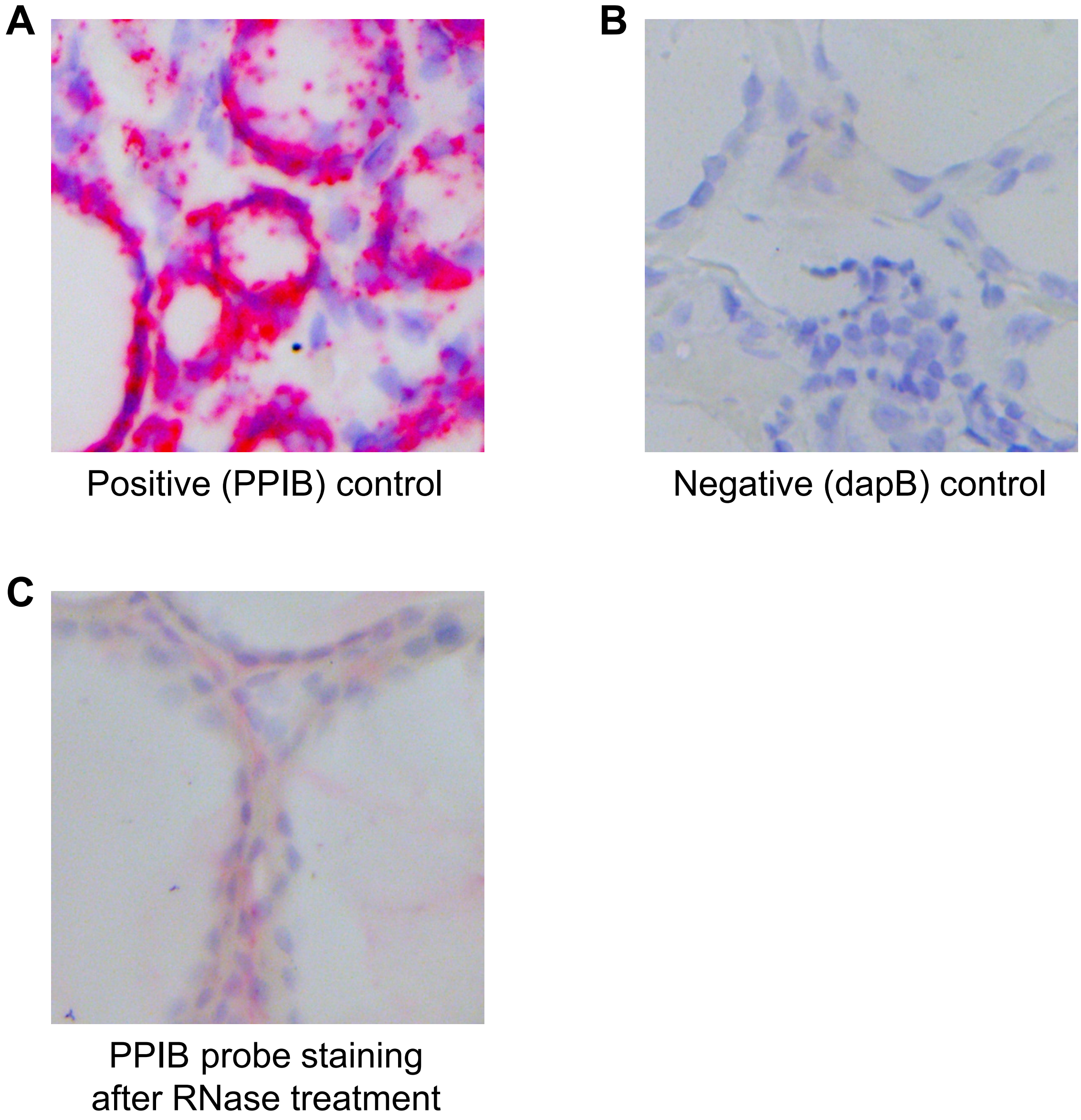

Fig S2. Representative photomicrographs of imprinted gene *GNAS* expression in normal, benign and malignant tumor samples

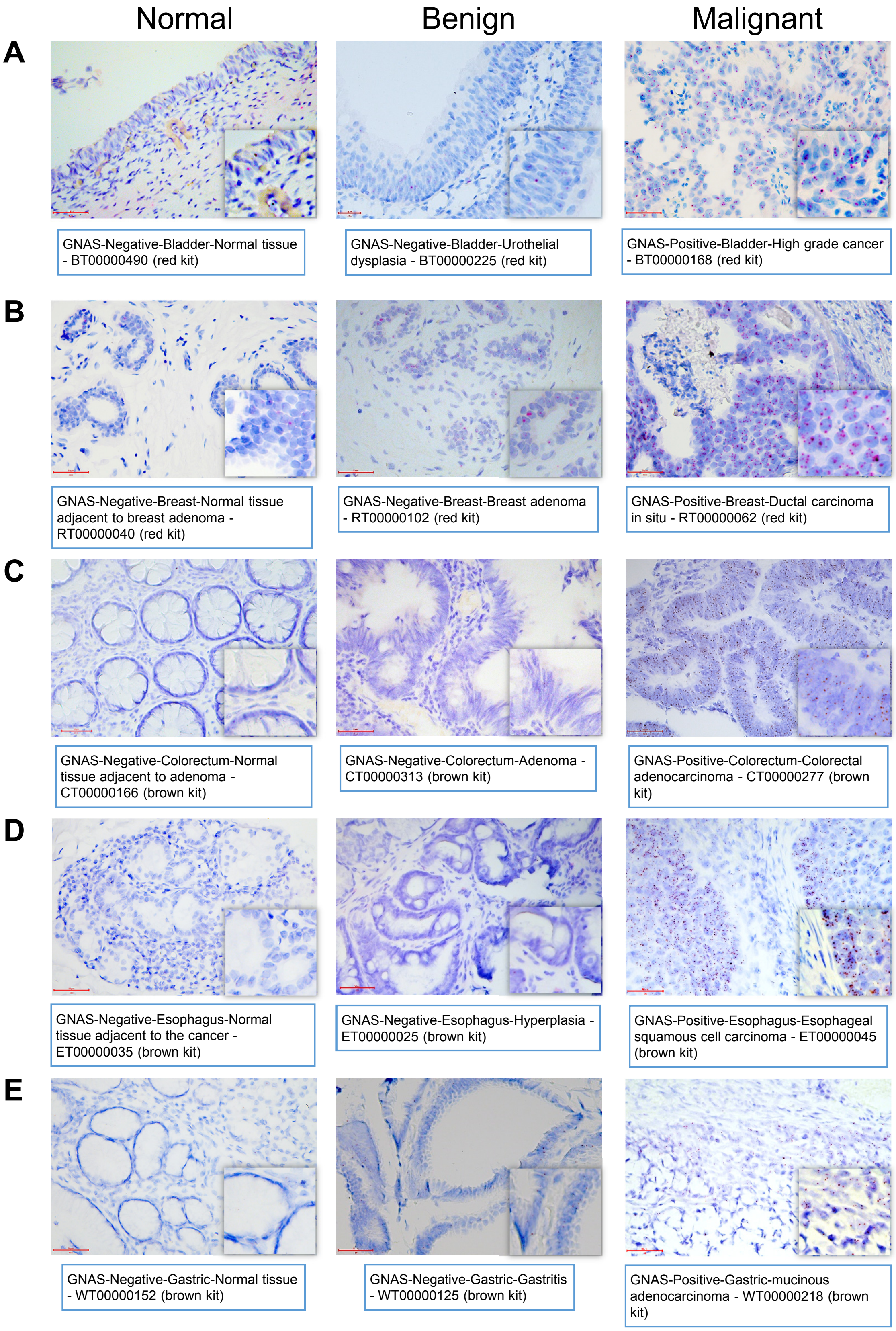

Different chromogenic kits (red or brown) produce different color of signals, but the results are the same.

Fig S2. Representative photomicrographs of imprinted gene *GNAS* expression in normal, benign and malignant tumor samples

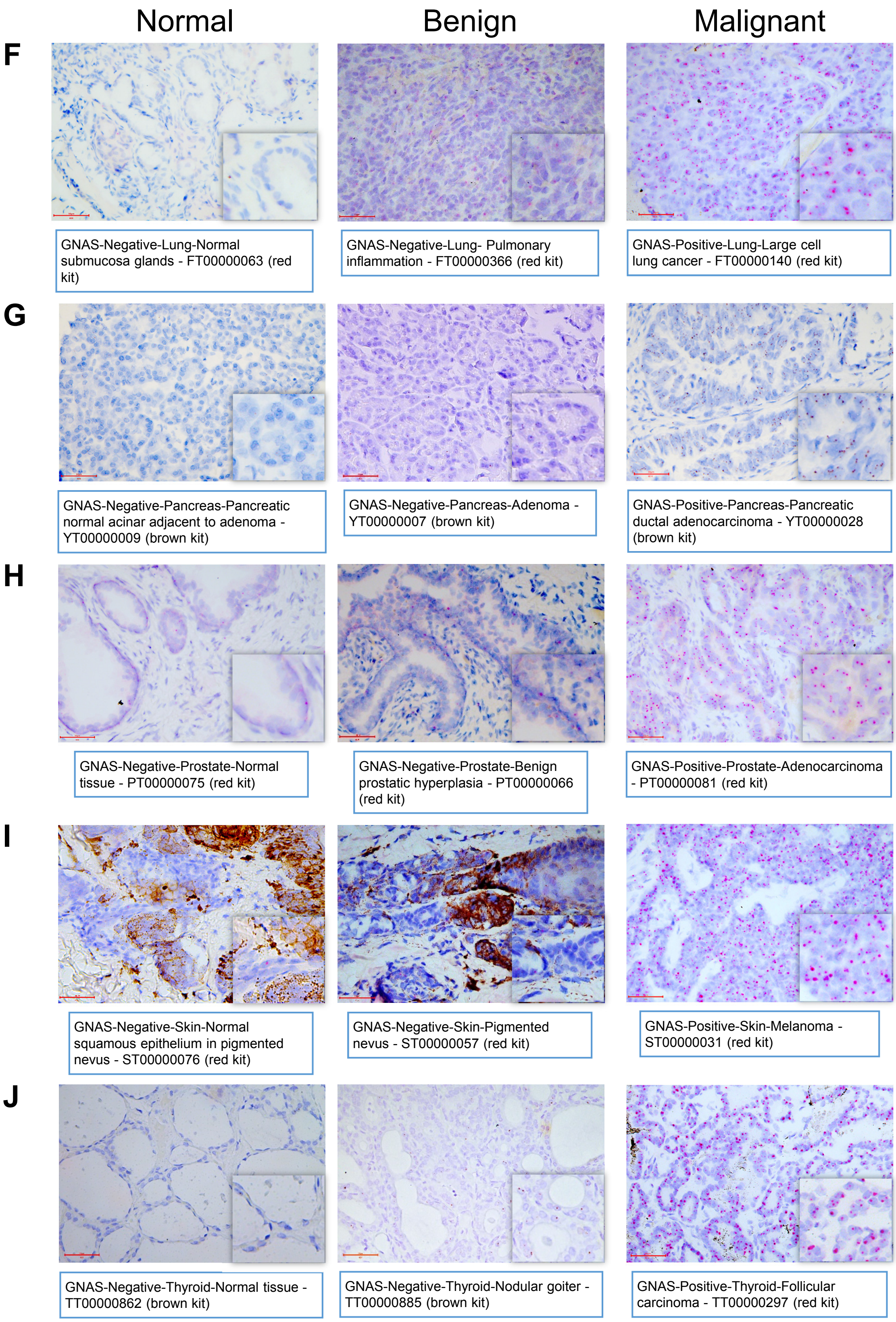

Different chromogenic kits (red or brown) produce different color of signals, but the results are the same.

Fig S3. Representative photomicrographs of imprinted gene *GRB10* expression in normal, benign and malignant tumor samples

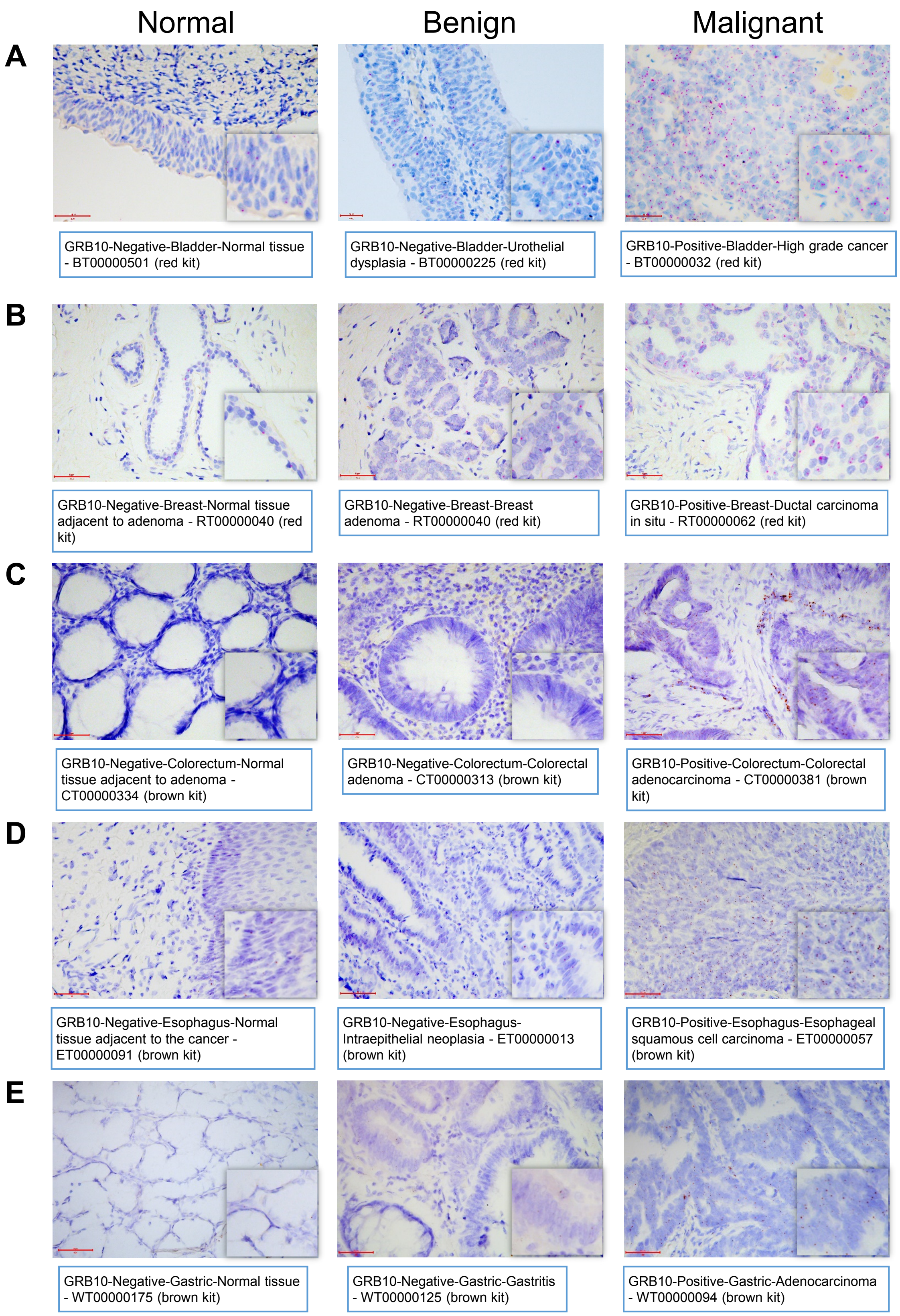

Different chromogenic kits (red or brown) produce different color of signals, but the results are the same.

Fig S3. Representative photomicrographs of imprinted gene *GRB10* expression in normal, benign and malignant tumor samples

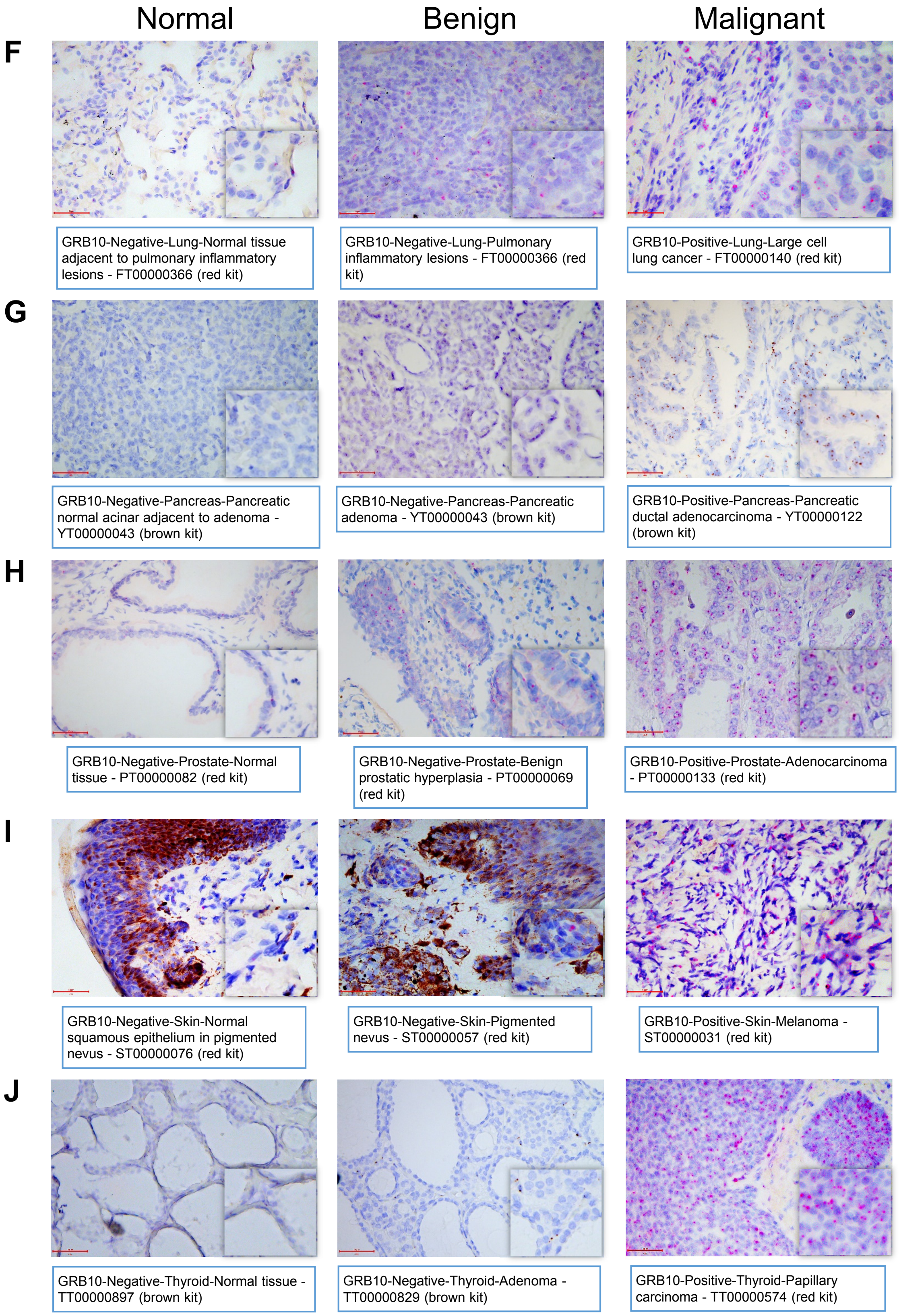

Different chromogenic kits (red or brown) produce different color of signals, but the results are the same.

Fig S4. Representative photomicrographs of imprinted gene *SNRPN* expression in normal, benign and malignant tumor samples

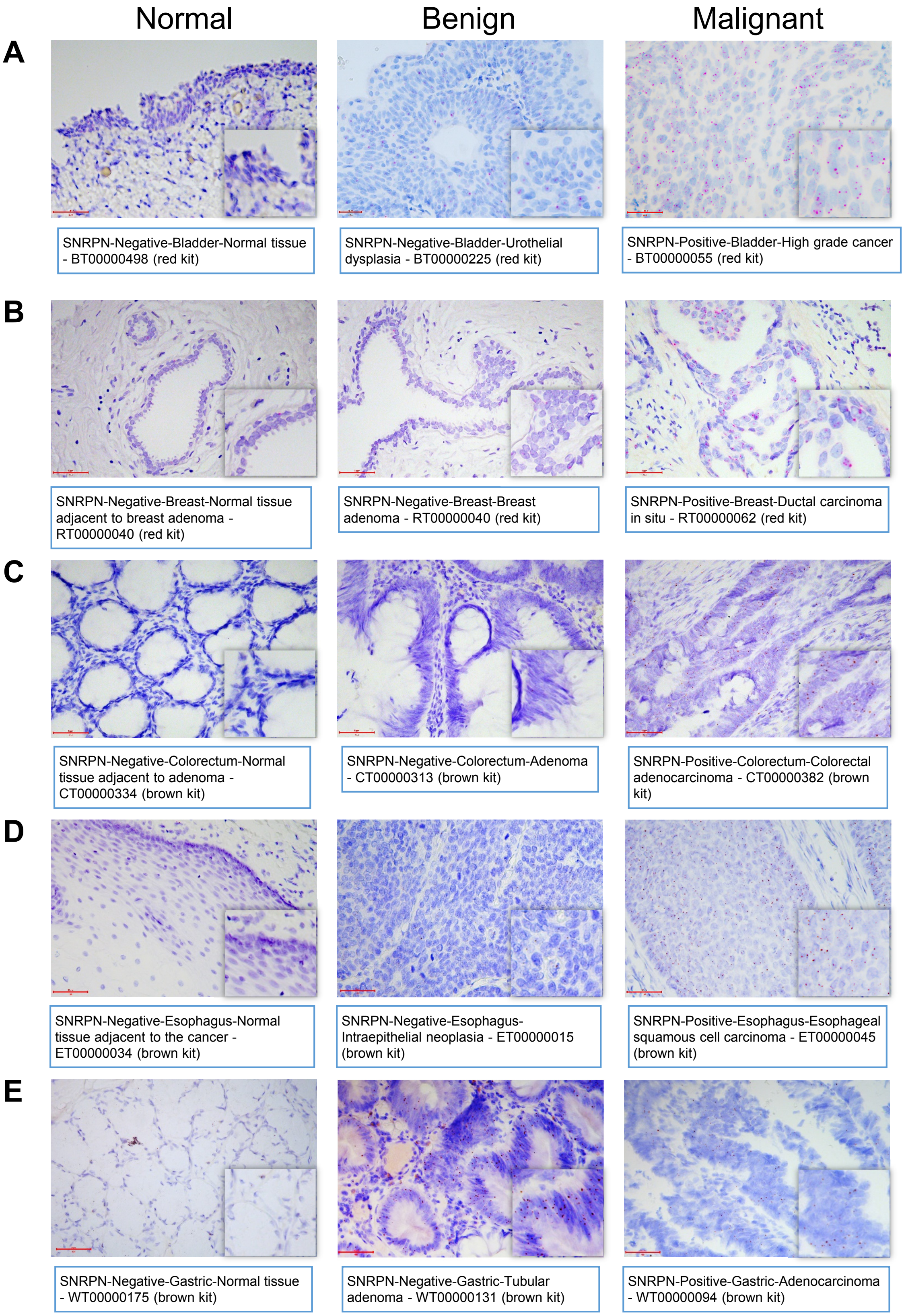

Different chromogenic kits (red or brown) produce different color of signals, but the results are the same.

Fig S4. Representative photomicrographs of imprinted gene *SNRPN* expression in normal, benign and malignant tumor samples

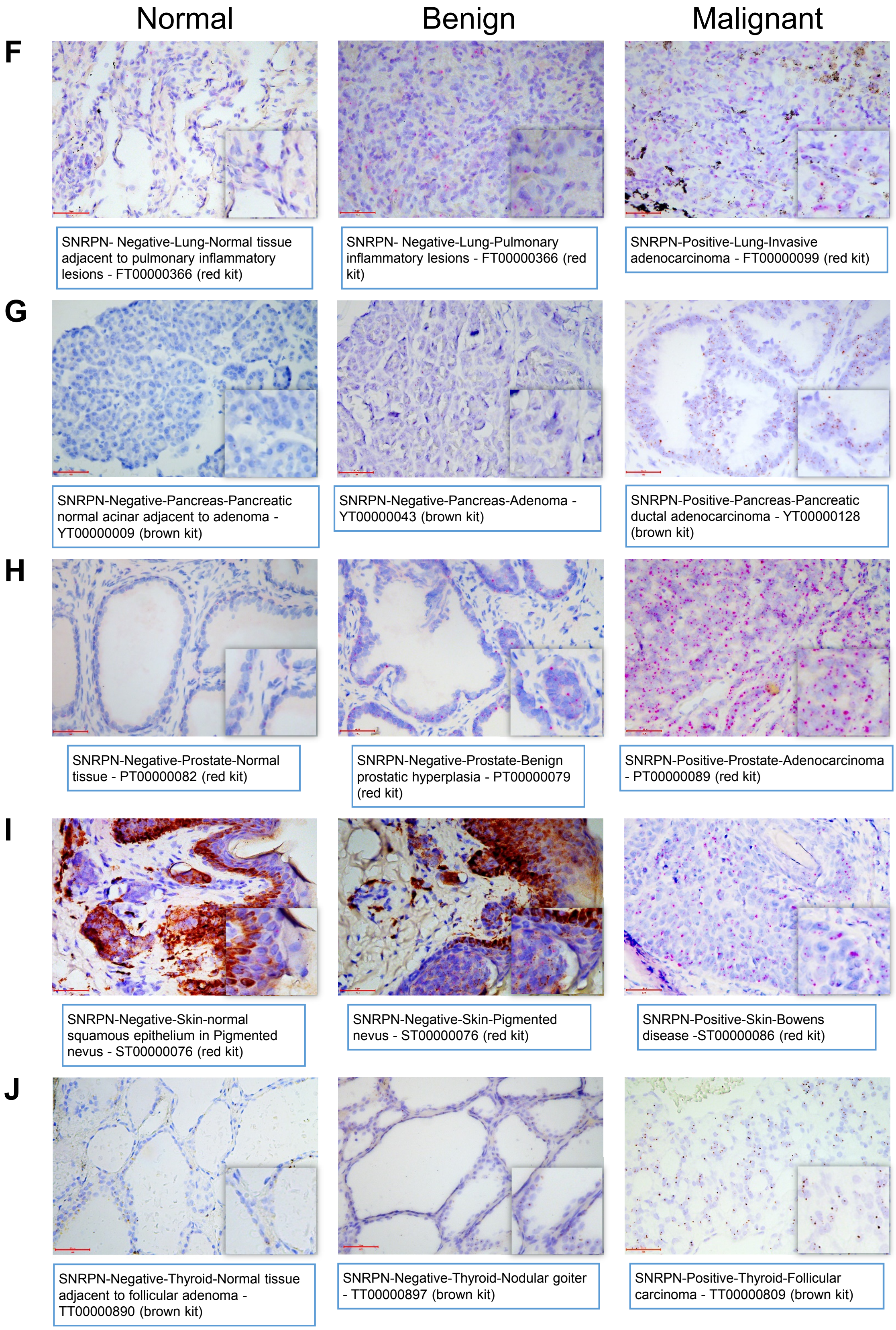

Different chromogenic kits (red or brown) produce different color of signals, but the results are the same.

Fig. S5. ROC curves for gene screening set.

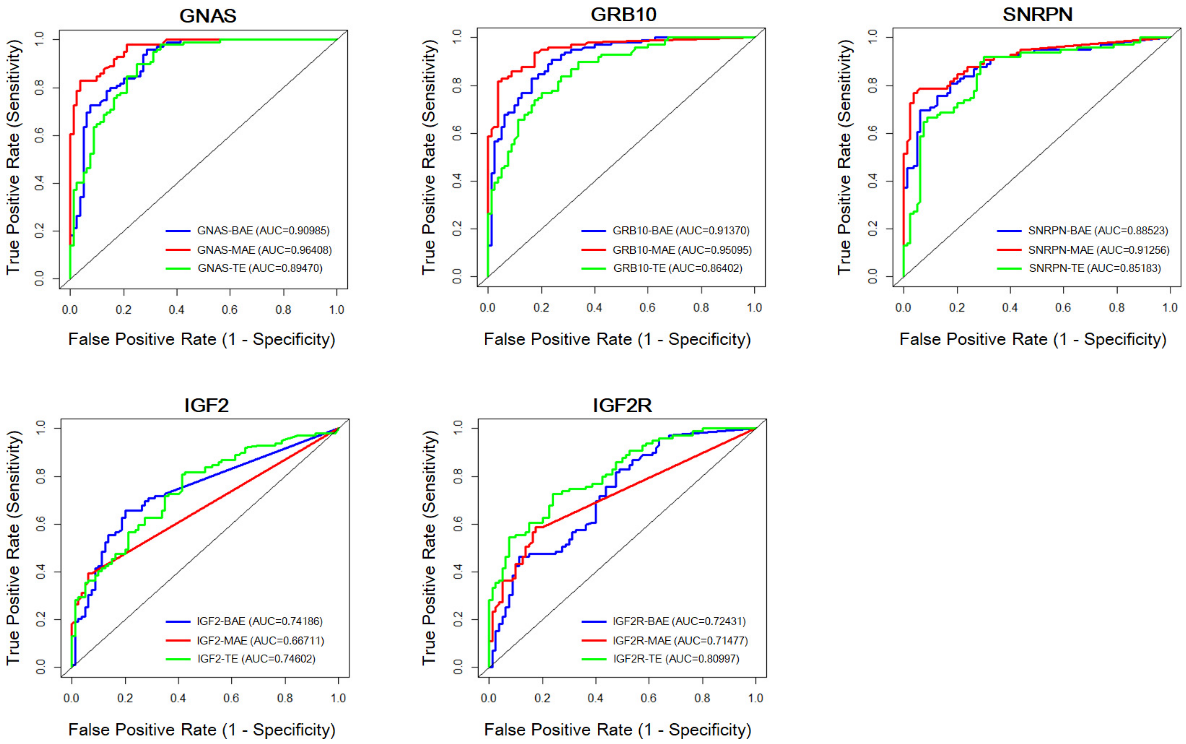

Fig. S6. ROC curves for cancer-specific diagnostic model building set.

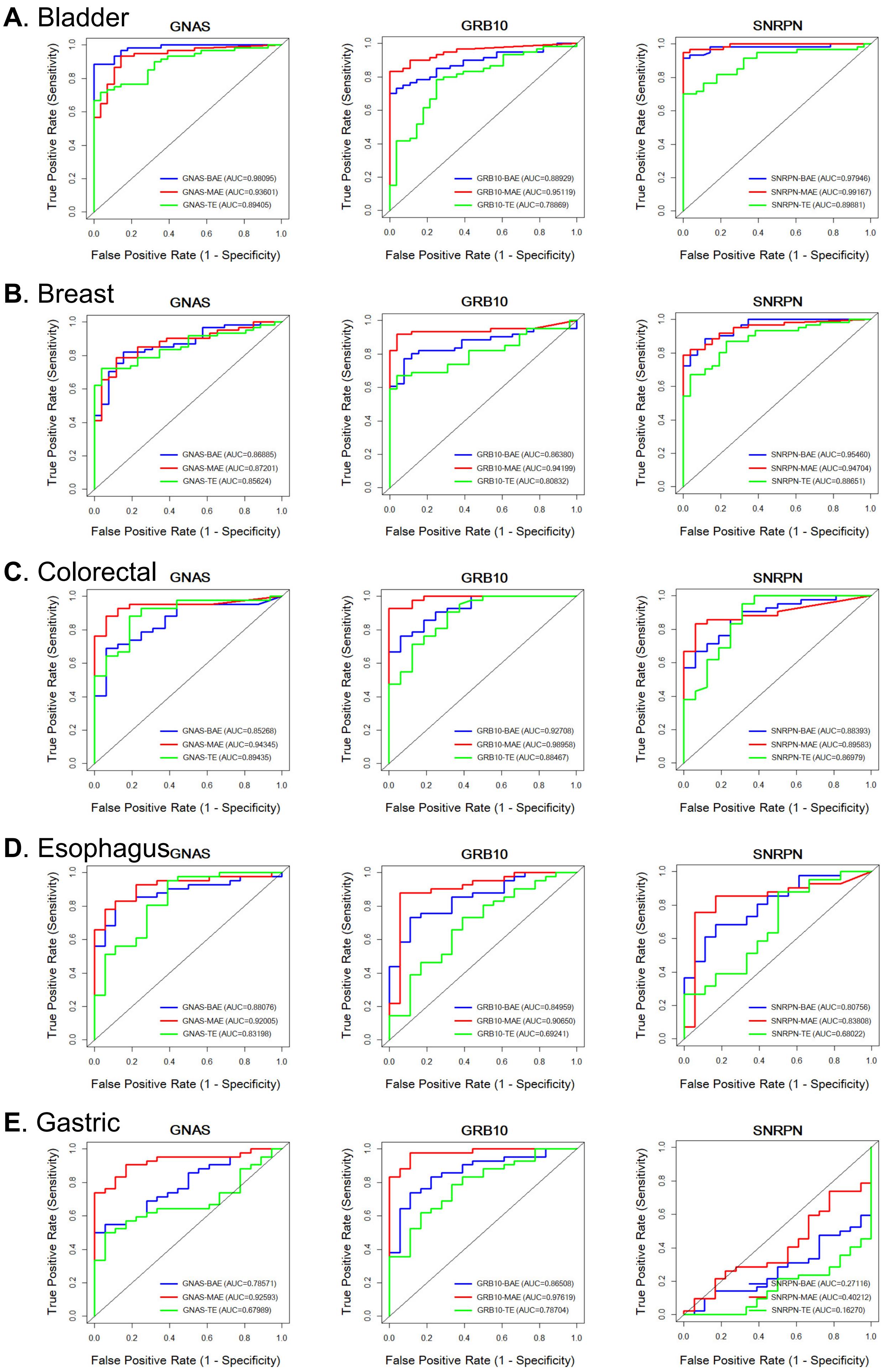

Fig. S6. ROC curves for cancer-specific diagnostic model building set.

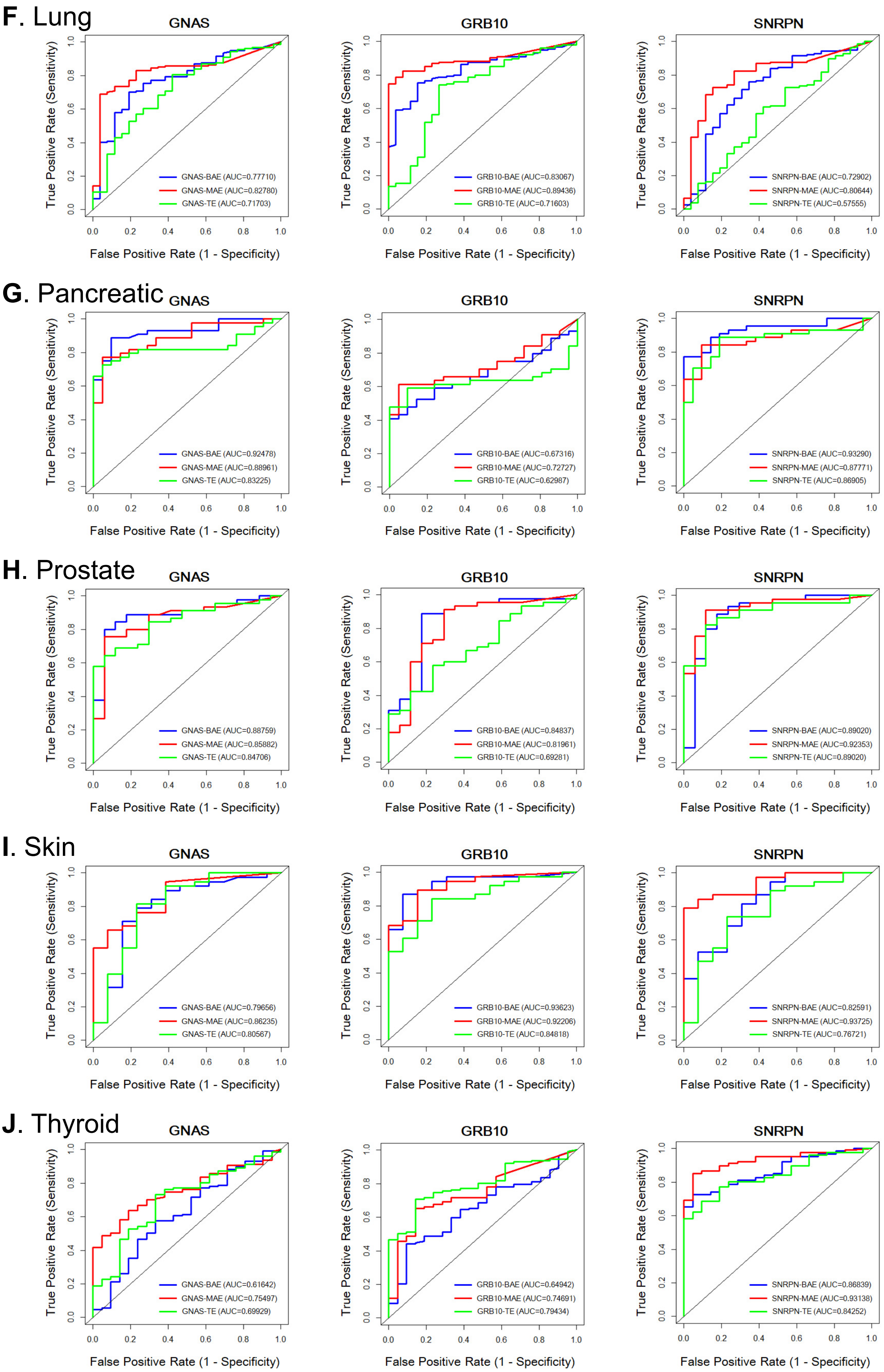

Supplement: Supplementary file 1 — Additional file 1. Supplementary Data [file 13148_2020_861_MOESM1_ESM.zip › MethodologyPaperSupplementaryFigures.pdf]
