# Supplementary material for: Novel visualized quantitative epigenetic imprinted gene biomarkers diagnose the malignancy of ten cancer types
Source: Clin Epigenetics. 2020 May 24;12:71. doi: 10.1186/s13148-020-00861-1 (PMC7245932; doi:10.1186/s13148-020-00861-1)
Supplement: Supplementary file 1 — Additional file 1. Supplementary Data [file 13148_2020_861_MOESM1_ESM.zip › MethodologyPaperSupplements.docx]

**Supplementary Data**

**Supplementary Methods**

**Images selection and cell counting**

Four high power fields with the most imprinting signals from each sample were selected under 400× microscope, with a total cell count usually from 1,200 to 2,000.

Technologists are trained and audited weekly to ensure technician consistency, with the tight 2% inter-observer variability.

**Supplementary Figure Legends**

**Fig S1.** Quality control verification process for the QCIGISH method. (A) Staining of positive control probe PPIB indicating RNA quality. (B) Staining of negative control probe indicating the presence or absence of non-specific signals. (C) Staining of positive control probe after RNase treatment.

**Fig S2.** Representative photomicrographs of imprinted gene GNAS expression in normal, benign and malignant tumor samples: (A) bladder, (B) breast, (C) colorectal, (D), esophagus, (E), gastric, (F), lung, (G), pancreatic, (H), prostate, (I), skin, (J), thyroid.

**Fig S3.** Representative photomicrographs of imprinted gene GNAS expression in normal, benign and malignant tumor samples: (A) bladder, (B) breast, (C) colorectal, (D), esophagus, (E), gastric, (F), lung, (G), pancreatic, (H), prostate, (I), skin, (J), thyroid.

**Fig S4.** Representative photomicrographs of imprinted gene GNAS expression in normal, benign and malignant tumor samples: (A) bladder, (B) breast, (C) colorectal, (D), esophagus, (E), gastric, (F), lung, (G), pancreatic, (H), prostate, (I), skin, (J), thyroid.

**Fig S5.** ROC curves for gene screening set.

**Fig S6.** ROC curves for cancer-specific diagnostic model building set. (A) bladder, (B) breast, (C) colorectal, (D), esophagus, (E), gastric, (F), lung, (G), pancreatic, (H), prostate, (I), skin, (J), thyroid.

**Supplementary Tables**

**Table S1. Comparative Statistical Analysis of *GNAS*, *GRB10*, *SNRPN*, *IGF2* and *IGF2R* Genes’ Allelic Expression Status between Normal, Benign and Malignant Cases using the Gene Screening Set for All Cancers.**

| ***GNAS* Expression Status Comparison**  **by Independent Groups** | | | | **Robust Rank Order Test Computed *p*-value** |
| --- | --- | --- | --- | --- |
| **Category** | **n** | **Median** | **IQR** |  |
| **BAE** | | | | |
| Normal | 30 | 1.51% | 6.25% | **0.00000***  (2.68e-09) |
| Benign | 50 | 9.31% | 9.13% |  |
| Normal | 30 | 1.51% | 6.25% | **0.00000***  (<2.20e-16) |
| Malignant | 99 | 20.80% | 10.00% |  |
| Benign | 50 | 9.31% | 9.13% | **0.00000***  (<2.20e-16) |
| Malignant | 99 | 20.80% | 10.00% |  |
| **MAE** | | | | |
| Normal | 30 | 0.00% | 0.00% | **0.00051*** |
| Benign | 50 | 0.79% | 1.54% |  |
| Normal | 30 | 0.00% | 0.00% | **0.00000***  (<2.20e-16) |
| Malignant | 99 | 6.25% | 7.27% |  |
| Benign | 50 | 0.79% | 1.54% | **0.00000***  (<2.20e-16) |
| Malignant | 99 | 6.25% | 7.27% |  |
| **TE** | | | | |
| Normal | 30 | 4.96% | 8.40% | **0.00000***  (3.08e-08) |
| Benign | 50 | 14.00% | 19.70% |  |
| Normal | 30 | 4.96% | 8.40% | **0.00000***  (<2.20e-16) |
| Malignant | 99 | 37.10% | 30.00% |  |
| Benign | 50 | 14.00% | 19.70% | **0.00000***  (<2.20e-16) |
| Malignant | 99 | 37.10% | 30.00% |  |
| ***GRB10* Expression Status Comparison**  **by Independent Groups** | | | | **Robust Rank Order Test Computed *p*-value** |
| **Category** | **n** | **Median** | **IQR** |  |
| **BAE** | | | | |
| Normal | 30 | 2.78% | 6.20% | **0.00005*** |
| Benign | 50 | 8.94% | 7.58% |  |
| Normal | 30 | 2.78% | 6.20% | **0.00000***  (<2.20e-16) |
| Malignant | 99 | 18.10% | 9.39% |  |
| Benign | 50 | 8.94% | 7.58% | **0.00000***  (2.23e-06) |
| Malignant | 99 | 18.10% | 9.39% |  |
| **MAE** | | | | |
| Normal | 30 | 0.00% | 0.00% | **0.00334*** |
| Benign | 50 | 0.43% | 1.63% |  |
| Normal | 30 | 0.00% | 0.00% | **0.00000***  (<2.20e-16) |
| Malignant | 99 | 5.60% | 6.39% |  |
| Benign | 50 | 0.43% | 1.63% | **0.00000***  (<2.20e-16) |
| Malignant | 99 | 5.60% | 6.39% |  |
| **TE** | | | | |
| Normal | 30 | 5.04% | 5.85% | **0.00005*** |
| Benign | 50 | 11.20% | 12.70% |  |
| Normal | 30 | 5.04% | 5.85% | **0.00000***  (<2.20e-16) |
| Malignant | 99 | 26.80% | 25.60% |  |
| Benign | 50 | 11.20% | 12.70% | **0.00000***  (<2.20e-16) |
| Malignant | 99 | 26.80% | 25.60% |  |
| ***SNRPN* Expression Status Comparison**  **by Independent Groups** | | | | **Robust Rank Order Test Computed *p*-value** |
| **Category** | **n** | **Median** | **IQR** |  |
| **BAE** | | | | |
| Normal | 27** | 2.08% | 6.32% | **0.00003*** |
| Benign | 45** | 7.14% | 8.10% |  |
| Normal | 27** | 2.08% | 6.32% | **0.00000***  (<2.20e-16) |
| Malignant | 89** | 20.60% | 7.63% |  |
| Benign | 45** | 7.14% | 8.10% | **0.00000***  (<2.20e-16) |
| Malignant | 89** | 20.60% | 7.63% |  |
| **MAE** | | | | |
| Normal | 27** | 0.00% | 0.00% | **0.00014*** |
| Benign | 45** | 0.58% | 1.58% |  |
| Normal | 27** | 0.00% | 0.00% | **0.00000***  (<2.20e-16) |
| Malignant | 89** | 5.32% | 7.15% |  |
| Benign | 45** | 0.58% | 1.58% | **0.00000***  (<2.20e-16) |
| Malignant | 89** | 5.32% | 7.15% |  |
| **TE** | | | | |
| Normal | 27** | 6.43% | 6.98% | **0.00000***  (1.06e-07) |
| Benign | 45** | 12.60% | 21.10% |  |
| Normal | 27** | 6.43% | 6.98% | **0.00000***  (<2.20e-16) |
| Malignant | 89** | 44.80% | 20.80% |  |
| Benign | 45** | 12.60% | 21.10% | **0.00000***  (<2.20e-16) |
| Malignant | 89** | 44.80% | 20.80% |  |
| ***IGF2* Expression Status Comparison**  **by Independent Groups** | | | | **Robust Rank Order Test Computed *p*-value** |
| **Category** | **n** | **Median** | **IQR** |  |
| **BAE** | | | | |
| Normal | 30 | 0.00% | 0.00% | 0.01783 |
| Benign | 50 | 0.00% | 4.62% |  |
| Normal | 30 | 0.00% | 0.00% | **0.00000***  (<2.21e-11) |
| Malignant | 99 | 6.11% | 10.10% |  |
| Benign | 50 | 0.00% | 4.62% | **0.00048*** |
| Malignant | 99 | 6.11% | 10.10% |  |
| **MAE** | | | | |
| Normal | 30 | 0.00% | 0.00% | 0.09585 |
| Benign | 50 | 0.00% | 0.00% |  |
| Normal | 30 | 0.00% | 0.00% | **0.00003*** |
| Malignant | 99 | 0.00% | 0.83% |  |
| Benign | 50 | 0.00% | 0.00% | **0.00483*** |
| Malignant | 99 | 0.00% | 0.83% |  |
| **TE** | | | | |
| Normal | 30 | 0.68% | 2.45% | **0.00422*** |
| Benign | 50 | 1.60% | 7.04% |  |
| Normal | 30 | 0.68% | 2.45% | **0.00000***  (<2.20e-16) |
| Malignant | 99 | 6.63% | 18.10% |  |
| Benign | 50 | 1.60% | 7.04% | **0.00000***  (4.74e-06) |
| Malignant | 99 | 6.63% | 18.10% |  |
| ***IGF2R* Expression Status Comparison**  **by Independent Groups** | | | | **Robust Rank Order Test Computed *p*-value** |
| **Category** | **n** | **Median** | **IQR** |  |
| **BAE** | | | | |
| Normal | 30 | 0.00% | 3.12% | **0.00006*** |
| Benign | 50 | 5.94% | 6.18% |  |
| Normal | 30 | 0.00% | 3.12% | **0.00000***  (8.22e-16) |
| Malignant | 99 | 8.02% | 11.10% |  |
| Benign | 50 | 5.94% | 6.18% | **0.00140*** |
| Malignant | 99 | 8.02% | 11.10% |  |
| **MAE** | | | | |
| Normal | 30 | 0.00% | 0.00% | **0.00765*** |
| Benign | 50 | 0.00% | 0.33% |  |
| Normal | 30 | 0.00% | 0.00% | **0.00000***  (1.64e-09) |
| Malignant | 99 | 0.40% | 1.54% |  |
| Benign | 50 | 0.00% | 0.33% | **0.00296*** |
| Malignant | 99 | 0.40% | 1.54% |  |
| **TE** | | | | |
| Normal | 30 | 2.31% | 5.39% | **0.00004*** |
| Benign | 50 | 9.02% | 13.40% |  |
| Normal | 30 | 2.31% | 5.39% | **0.00000***  (<2.20e-16) |
| Malignant | 99 | 20.80% | 24.10% |  |
| Benign | 50 | 9.02% | 13.40% | **0.00000***  (2.37e-10) |
| Malignant | 99 | 20.80% | 24.10% |  |

* Significant at alpha = 0.01

** Gastric samples were excluded from the analysis for SNRPN gene

**Table S2. BAE, MAE and TE Threshold Analysis for the Gene Screening Set.**

| **Comparison of Optimal ROC Threshold Values for All Cancer Types** | | | | |
| --- | --- | --- | --- | --- |
| **Genes** | **Expression** | **Optimal Threshold** | **Sensitivity** | **Specificity** |
| *GNAS* | BAE | 14.69% | 79.80% | 85.00% |
|  | MAE | 1.80% | 87.88% | 87.50% |
|  | TE | 22.88% | 84.85% | 78.75% |
| *GRB10* | BAE | 11.81% | 82.83% | 83.75% |
|  | MAE | 2.33% | 85.86% | 91.25% |
|  | TE | 15.72% | 76.77% | 80.00% |
| *SNRPN* | BAE | 13.40% | 80.81% | 82.50% |
|  | MAE | 2.35% | 78.79% | 93.75% |
|  | TE | 19.24% | 89.90% | 71.25% |
| *IGF2* | BAE | 3.33% | 65.66% | 80.00% |
|  | MAE | 0.15% | 39.39% | 93.75% |
|  | TE | 2.95% | 71.72% | 65.00% |
| *IGF2R* | BAE | 4.82% | 71.72% | 58.75% |
|  | MAE | 0.24% | 58.59% | 82.50% |
|  | TE | 10.92% | 72.73% | 76.25% |

**Table S3. Primary Negative/Positive Classification Models for Imprinted Genes.**

| **Genes** | | ***GNAS*** | ***GRB10*** | ***SNRPN**** | ***IGF2*** | ***IGF2R*** |
| --- | --- | --- | --- | --- | --- | --- |
| **Adjusted**  **Thresholds** | BAE | 14.00% | 11.00% | 11.00% | 3.00% | 8.00% |
|  | MAE | 2.50% | 1.50% | 2.30% | 0.10% | 0.40% |
|  | TE | 10.00% | 9.00% | 10.00% | 3.00% | 6.00% |
| **Sensitivity** | | 88.89% | 88.89% | 96.63% | 58.59% | 54.55% |
| **Specificity** | | 82.50% | 82.50% | 80.56% | 81.25% | 80.00% |

* Gastric samples were excluded from sensitivity and specificity calculation for SNRPN gene.

**Table S4. Comparative Statistical Analysis of GNAS, GRB10 and SNRPN Genes’ Allelic Expression Status between Benign and Malignant Cases using the Diagnostic Model Building Set for each Cancer Type.**

|  | **Gene** | ***GNAS*** | | | ***GRB10*** | | | ***SNRPN*** | | |
| --- | --- | --- | --- | --- | --- | --- | --- | --- | --- | --- |
| **Cancer type** | **Expression** | **Benign** | **Malignant** | **Robust Rank Order Test**  **Computed**  ***p*-value** | **Benign** | **Malignant** | **Robust Rank Order Test**  **Computed**  ***p*-value** | **Benign** | **Malignant** | **Robust Rank Order Test**  **Computed**  ***p*-value** |
|  |  | **n**  **Median**  **(IQR)** | **n**  **Median**  **(IQR)** |  | **n**  **Median**  **(IQR)** | **n**  **Median**  **(IQR)** |  | **n**  **Median**  **(IQR)** | **n**  **Median**  **(IQR)** |  |
| **Bladder** | BAE | 28  7.77% (4.65%) | 60  19.80% (8.33%) | **0.00000***  (<2.20e-16) | 28  7.96% (4.86%) | 60  17.10% (7.82%) | **0.00000***  (<2.20e-16) | 28  8.52% (4.10%) | 60  23.50% (8.33%) | **0.00000***  (<2.20e-16) |
|  | MAE | 28  0.55% (1.23%) | 60  5.68% (5.80%) | **0.00000***  (<2.20e-16) | 28  0.00% (1.02%) | 60  4.43% (5.84%) | **0.00000***  (<2.20e-16) | 28  0.43% (1.27%) | 60  11.60% (15.00%) | **0.00000***  (<2.20e-16) |
|  | TE | 28  10.40% (10.90%) | 60  34.20% (24.80%) | **0.00000***  (<2.20e-16) | 28  14.50% (8.13%) | 60  24.90% (15.80%) | **0.00000***  (<2.20e-16) | 28  11.30% (14.00%) | 60  54.90% (36.50%) | **0.00000***  (<2.20e-16) |
| **Breast** | BAE | 26  12.90% (6.08%) | 61  22.60% (7.58%) | **0.00000***  (<2.20e-16) | 26  9.06% (4.09%) | 61  20.40% (11.30%) | **0.00000***  (<2.20e-16) | 26  7.76% (6.63%) | 61  22.90% (9.07%) | **0.00000***  (<2.20e-16) |
|  | MAE | 26  1.71% (1.71%) | 61  9.02% (9.02%) | **0.00000***  (<2.20e-16) | 26  0.49% (1.02%) | 61  7.86% (13.10%) | **0.00000***  (<2.20e-16) | 26  0.37% (1.14%) | 61  5.53% (9.30%) | **0.00000***  (<2.20e-16) |
|  | TE | 26  26.30% (18.00%) | 61  51.90% (33.70%) | **0.00000***  (<2.20e-16) | 26  16.60% (11.00%) | 61  39.40% (34.00%) | **0.00000***  (6.44e-12) | 26  23.30% (14.50%) | 61  61.20% (33.60%) | **0.00000***  (<2.20e-16) |
| **Colorectal** | BAE | 16  11.60% (15.20%) | 42  21.00% (8.91%) | **0.00000***  (6.28e-11) | 16  4.92% (11.80%) | 42  20.50% (9.42%) | **0.00000***  (<2.20e-16) | 16  10.40% (8.49%) | 42  21.80% (9.11%) | **0.00000***  (<2.20e-16) |
|  | MAE | 16  0.74% (1.06%) | 42  8.35% (10.10%) | **0.00000***  (<2.20e-16) | 16  0.00% (0.81%) | 42  11.90% (11.60%) | **0.00000***  (<2.20e-16) | 16  0.19% (1.28%) | 42  5.10% (5.22%) | **0.00000***  (<2.15e-14) |
|  | TE | 16  7.20% (9.32%) | 42  44.80% (36.00%) | **0.00000***  (<2.20e-16) | 16  4.30% (8.93%) | 42  31.60% (36.30%) | **0.00000***  (2.42e-15) | 16  10.20% (17.80%) | 42  36.20% (24.00%) | **0.00000***  (4.02e-11) |
| **Esophagus** | BAE | 18  13.30% (7.25%) | 41  25.40% (6.19%) | **0.00000***  (<2.20e-16) | 18  13.50% (5.14%) | 41  20.80% (7.84%) | **0.00000***  (3.99e-12) | 18  14.40% (8.36%) | 41  22.80% (10.70%) | **0.00000***  (9.05e-08) |
|  | MAE | 18  1.08% (1.46%) | 41  10.70% (12.50%) | **0.00000***  (<2.20e-16) | 18  1.65% (1.86%) | 41  10.80% (9.53%) | **0.00000***  (<2.20e-16) | 18  1.32% (1.63%) | 41  6.16% (6.68%) | **0.00000***  (4.90e-08) |
|  | TE | 18  15.80% (19.30%) | 41  43.90% (26.40%) | **0.00000***  (1.64e-08) | 18  21.10% (22.50%) | 41  32.20% (17.70%) | **0.00610*** | 18  27.90% (28.80%) | 41  40.80% (25.30%) | 0.01096 |
| **Gastric** | BAE | 18  13.20% (6.26%) | 42  19.80% (12.50%) | **0.00000***  (8.52e-07) | 18  10.90% (4.90%) | 42  20.20% (8.03%) | **0.00000***  (9.25e-14) | 18**  24.10% (8.32%) | 42**  16.80% (11.50%) | 0.99980 |
|  | MAE | 18  1.48% (1.78%) | 42  6.15% (7.19%) | **0.00000***  (<2.20e-16) | 18  1.04% (1.13%) | 42  7.48% (4.80%) | **0.00000***  (<2.20e-16) | 18**  5.56% (4.90%) | 42**  3.91% (6.62%) | 0.89680 |
|  | TE | 18  26.20% (7.13%) | 42  39.50% (33.30%) | **0.00487*** | 18  14.30% (13.80%) | 42  29.60% (21.20%) | **0.00000***  (2.14e-06) | 18**  52.60% (26.10%) | 42**  30.20% (23.30%) | 1.00000 |
| **Lung** | BAE | 26  9.77% (7.06%) | 154  18.20% (13.40%) | **0.00000***  (3.50e-09) | 26  9.93% (7.18%) | 154  20.10% (11.70%) | **0.00000***  (<2.20e-16) | 26  9.02% (8.65%) | 154  17.50% (11.90%) | **0.00004*** |
|  | MAE | 26  0.79% (1.39%) | 154  5.14% (8.47%) | **0.00000***  (9.47e-15) | 26  1.12% (1.62%) | 154  9.07% (13.40%) | **0.00000***  (<2.20e-16) | 26  0.53% (1.40%) | 154  4.39% (7.34%) | **0.00000***  (1.42e-10) |
|  | TE | 26  16.10% (21.60%) | 154  33.10% (26.60%) | **0.00004*** | 26  13.50% (14.80%) | 154  28.80% (28.10%) | **0.00000***  (9.87e-05) | 26  27.10% (24.60%) | 154  32.30% (26.40%) | 0.11850 |
| **Pancreatic** | BAE | 21  13.00% (4.50%) | 44  23.10% (8.45%) | **0.00000***  (<2.20e-16) | 21  16.10% (3.43%) | 44  20.50% (13.50%) | **0.00437*** | 21  7.98% (1.89%) | 44  20.50% (10.10%) | **0.00000***  (<2.20e-16) |
|  | MAE | 21  2.25% (1.16%) | 44  7.82% (14.40%) | **0.00000***  (<2.20e-16) | 21  3.25% (3.14%) | 44  9.64% (18.10%) | **0.00018*** | 21  0.65% (0.84%) | 44  4.87% (8.28%) | **0.00000***  (5.16e-16) |
|  | TE | 21  21.80% (4.97%) | 44  50.80% (45.40%) | **0.00000***  (7.35e-11) | 21  31.30% (10.20%) | 44  46.50% (49.40%) | 0.032920 | 21  17.00% (8.73%) | 44  43.02% (21.85%) | **0.00000***  (1.10e-15) |
| **Prostate** | BAE | 17  9.46% (4.83%) | 45  20.60% (9.06%) | **0.00000***  (<2.20e-16) | 17  8.39% (3.83%) | 45  15.50% (7.07%) | **0.00000***  (9.09e-09) | 17  10.60% (3.71%) | 45  25.40% (8.56%) | **0.00000***  (2.36e-12) |
|  | MAE | 17  1.01% (1.70%) | 45  4.02% (5.79%) | **0.00000***  (1.40e-10) | 17  0.52% (1.98%) | 45  3.04% (4.21%) | **0.00000***  (2.84e-06) | 17  0.88% (2.02%) | 45  8.97% (11.00%) | **0.00000***  (<2.20e-16) |
|  | TE | 17  14.80% (13.10%) | 45  35.20% (27.80%) | **0.00000***  (1.40e-12) | 17  15.90% (7.26%) | 45  20.60% (16.00%) | **0.00376*** | 17  20.20% (12.40%) | 45  57.50% (31.10%) | **0.00000***  (<2.20e-16) |
| **Skin** | BAE | 13  6.62% (5.99%) | 38  14.70% (5.51%) | **0.00016*** | 13  6.93% (6.45%) | 38  17.00% (6.23%) | **0.00000***  (<2.20e-16) | 13  10.80% (9.34%) | 38  18.20% (9.75%) | **0.00000***  (1.02e-06) |
|  | MAE | 13  0.00% (1.69%) | 38  2.70% (2.32%) | **0.00000***  (2.35e-09) | 13  0.00% (1.49%) | 38  5.74% (6.28%) | **0.00000***  (<2.20e-16) | 13  0.35% (1.57%) | 38  3.80% (4.25%) | **0.00000***  (<2.20e-16) |
|  | TE | 13  9.78% (12.40%) | 38  24.20% (15.60%) | **0.00009*** | 13  6.53% (5.18%) | 38  13.40% (10.00%) | **0.00000***  (4.57e-10) | 13  23.30% (13.80%) | 38  45.20% (22.30%) | **0.00046*** |
| **Thyroid** | BAE | 21  21.30% (8.74%) | 127  23.70% (7.44%) | 0.04384 | 21  11.20% (7.58%) | 127  15.20% (9.79%) | **0.00546*** | 21  14.40% (7.37%) | 127  22.20% (8.84%) | **0.00000***  (<2.20e-16) |
|  | MAE | 21  3.81% (2.90%) | 127  7.87% (11.00%) | **0.00000***  (7.25e-09) | 21  0.99% (1.22%) | 127  2.66% (4.11%) | **0.00000***  (1.78e-05) | 21  1.09% (1.04%) | 127  5.24% (6.30%) | **0.00000***  (<2.20e-16) |
|  | TE | 21  29.30% (20.10%) | 127  45.00% (27.70%) | **0.00038*** | 21  6.06% (4.73%) | 127  14.80% (18.40%) | **0.00000***  (1.69e-12) | 21  32.00% (19.20%) | 127  51.30% (28.00%) | **0.00000***  (<2.20e-16) |

* Significant at alpha = 0.01

** In gastric cancer, BAE, MAE and TE are higher in benign than malignant samples.

**Table S5. Bladder Cancer BAE, MAE and TE Threshold Analysis for the Model Building Set.**

| **Comparison of Optimal ROC Threshold Values for Bladder Cancer** | | | | |
| --- | --- | --- | --- | --- |
| **Genes** | **Expression** | **Optimal Threshold** | **Sensitivity** | **Specificity** |
| *GNAS* | BAE | 13.45% | 88.33% | 100.00% |
|  | MAE | 1.89% | 93.33% | 85.71% |
|  | TE | 20.38% | 75.00% | 89.29% |
| *GRB10* | BAE | 12.62% | 76.67% | 89.29% |
|  | MAE | 1.89% | 90.00% | 89.29% |
|  | TE | 17.71% | 78.33% | 75.00% |
| *SNRPN* | BAE | 15.47% | 93.33% | 96.43% |
|  | MAE | 2.49% | 96.67% | 96.43% |
|  | TE | 26.60% | 81.67% | 82.14% |

**Table S6. Breast Cancer BAE, MAE and TE Threshold Analysis for the Model Building Set.**

| **Comparison of Optimal ROC Threshold Values for Breast Cancer** | | | | |
| --- | --- | --- | --- | --- |
| **Genes** | **Expression** | **Optimal Threshold** | **Sensitivity** | **Specificity** |
| *GNAS* | BAE | 16.38% | 81.97% | 84.62% |
|  | MAE | 4.08% | 78.69% | 88.46% |
|  | TE | 41.10% | 72.13% | 96.15% |
| *GRB10* | BAE | 13.03% | 80.33% | 88.46% |
|  | MAE | 3.03% | 91.80% | 96.15% |
|  | TE | 25.51% | 67.21% | 96.15% |
| *SNRPN* | BAE | 15.04% | 88.53% | 88.46% |
|  | MAE | 2.67% | 81.97% | 96.15% |
|  | TE | 33.23% | 86.89% | 76.92% |

**Table S7. Colorectal Cancer BAE, MAE and TE Threshold Analysis for the Model Building Set.**

| **Comparison of Optimal ROC Threshold Values for Colorectal Cancer** | | | | |
| --- | --- | --- | --- | --- |
| **Genes** | **Expression** | **Optimal Threshold** | **Sensitivity** | **Specificity** |
| *GNAS* | BAE | 18.15% | 71.43% | 87.50% |
|  | MAE | 2.96% | 88.10% | 93.75% |
|  | TE | 19.47% | 88.10% | 81.25% |
| *GRB10* | BAE | 12.57% | 85.71% | 81.25% |
|  | MAE | 2.95% | 92.86% | 100.00% |
|  | TE | 17.34% | 76.19% | 81.25% |
| *SNRPN* | BAE | 13.71% | 85.71% | 75.00% |
|  | MAE | 2.01% | 83.33% | 93.75% |
|  | TE | 21.15% | 83.33% | 75.00% |

**Table S8. Esophagus Cancer BAE, MAE and TE Threshold Analysis for the Model Building Set.**

| **Comparison of Optimal ROC Threshold Values for Esophagus Cancer** | | | | |
| --- | --- | --- | --- | --- |
| **Genes** | **Expression** | **Optimal Threshold** | **Sensitivity** | **Specificity** |
| *GNAS* | BAE | 18.96% | 82.93% | 88.89% |
|  | MAE | 4.10% | 82.93% | 88.89% |
|  | TE | 24.79% | 80.49% | 72.22% |
| *GRB10* | BAE | 17.59% | 73.17% | 88.89% |
|  | MAE | 3.82% | 87.81% | 94.44% |
|  | TE | 73.17% | 73.17% | 61.11% |
| *SNRPN* | BAE | 18.64% | 68.29% | 83.33% |
|  | MAE | 2.28% | 85.37% | 83.33% |
|  | TE | 23.76% | 87.81% | 50.00% |

**Table S9. Gastric Cancer BAE, MAE and TE Threshold Analysis for the Model Building Set.**

| **Comparison of Optimal ROC Threshold Values for Gastric Cancer** | | | | |
| --- | --- | --- | --- | --- |
| **Genes** | **Expression** | **Optimal Threshold** | **Sensitivity** | **Specificity** |
| *GNAS* | BAE | 15.62% | 69.05% | 72.22% |
|  | MAE | 2.74% | 90.48% | 83.33% |
|  | TE | 32.85% | 57.14% | 83.33% |
| *GRB10* | BAE | 13.79% | 83.33% | 77.78% |
|  | MAE | 2.40% | 97.62% | 88.89% |
|  | TE | 21.85% | 78.57% | 66.67% |
| *SNRPN* | BAE | NA | NA | NA |
|  | MAE | NA | NA | NA |
|  | TE | NA | NA | NA |

**Table S10. Lung Cancer BAE, MAE and TE Threshold Analysis for the Model Building Set.**

| **Comparison of Optimal ROC Threshold Values for Lung Cancer** | | | | |
| --- | --- | --- | --- | --- |
| **Genes** | **Expression** | **Optimal Threshold** | **Sensitivity** | **Specificity** |
| *GNAS* | BAE | 12.50% | 70.13% | 80.77% |
|  | MAE | 1.43% | 83.12% | 76.92% |
|  | TE | 16.45% | 80.52% | 57.69% |
| *GRB10* | BAE | 13.90% | 75.33% | 84.62% |
|  | MAE | 2.84% | 82.47% | 92.31% |
|  | TE | 18.16% | 74.03% | 73.08% |
| *SNRPN* | BAE | 12.40% | 71.43% | 69.23% |
|  | MAE | 1.78% | 72.73% | 84.62% |
|  | TE | 27.93% | 61.04% | 57.69% |

**Table S11. Pancreatic Cancer BAE, MAE and TE Threshold Analysis for the Model Building Set.**

| **Comparison of Optimal ROC Threshold Values for Pancreatic Cancer** | | | | |
| --- | --- | --- | --- | --- |
| **Genes** | **Expression** | **Optimal Threshold** | **Sensitivity** | **Specificity** |
| *GNAS* | BAE | 15.96% | 88.64% | 90.48% |
|  | MAE | 4.56% | 77.27% | 95.24% |
|  | TE | 26.94% | 75.00% | 90.48% |
| *GRB10* | BAE | 18.28% | 59.09% | 76.19% |
|  | MAE | 7.39% | 61.36% | 95.24% |
|  | TE | 42.13% | 59.09% | 90.48% |
| *SNRPN* | BAE | 11.52% | 88.64% | 85.71% |
|  | MAE | 1.35% | 84.09% | 90.48% |
|  | TE | 20.03% | 88.64% | 80.95% |

**Table S12. Prostate Cancer BAE, MAE and TE Threshold Analysis for the Model Building Set.**

| **Comparison of Optimal ROC Threshold Values for Prostate Cancer** | | | | |
| --- | --- | --- | --- | --- |
| **Genes** | **Expression** | **Optimal Threshold** | **Sensitivity** | **Specificity** |
| *GNAS* | BAE | 13.13% | 84.44% | 88.24% |
|  | MAE | 2.02% | 75.56% | 94.12% |
|  | TE | 25.00% | 68.89% | 88.24% |
| *GRB10* | BAE | 10.68% | 88.89% | 82.35% |
|  | MAE | 1.45% | 91.11% | 70.59% |
|  | TE | 19.20% | 57.78% | 76.47% |
| *SNRPN* | BAE | 14.16% | 88.89% | 82.35% |
|  | MAE | 3.03% | 91.11% | 88.24% |
|  | TE | 39.11% | 82.22% | 88.24% |

**Table S13. Skin Cancer BAE, MAE and TE Threshold Analysis for the Model Building Set.**

| **Comparison of Optimal ROC Threshold Values for Skin Cancer** | | | | |
| --- | --- | --- | --- | --- |
| **Genes** | **Expression** | **Optimal Threshold** | **Sensitivity** | **Specificity** |
| *GNAS* | BAE | 11.41% | 78.95% | 76.92% |
|  | MAE | 1.70% | 76.32% | 76.92% |
|  | TE | 16.51% | 81.58% | 76.92% |
| *GRB10* | BAE | 12.80% | 86.84% | 92.31% |
|  | MAE | 1.91% | 89.47% | 84.62% |
|  | TE | 8.97% | 84.21% | 76.92% |
| *SNRPN* | BAE | 14.89% | 81.58% | 69.23% |
|  | MAE | 1.81% | 84.21% | 92.31% |
|  | TE | 31.70% | 73.68% | 76.92% |

**Table S14. Thyroid Cancer BAE, MAE and TE Threshold Analysis for the Model Building Set.**

| **Comparison of Optimal ROC Threshold Values for Thyroid Cancer** | | | | |
| --- | --- | --- | --- | --- |
| **Genes** | **Expression** | **Optimal Threshold** | **Sensitivity** | **Specificity** |
| *GNAS* | BAE | 22.35% | 57.48% | 66.67% |
|  | MAE | 5.06% | 66.93% | 76.19% |
|  | TE | 32.09% | 73.23% | 66.67% |
| *GRB10* | BAE | 12.09% | 64.57% | 61.91% |
|  | MAE | 1.51% | 65.35% | 85.71% |
|  | TE | 9.82% | 70.87% | 85.71% |
| *SNRPN* | BAE | 18.93% | 72.44% | 95.24% |
|  | MAE | 2.61% | 85.04% | 95.24% |
|  | TE | 37.66% | 77.17% | 80.95% |

**Table S15. Diagnostic Model Thresholds of Imprinted Genes GNAS, GRB10 and SNRPN for the Ten Cancers.**

|  | ***GNAS* Thresholds** | | | ***GRB10* Thresholds** | | | ***SNRPN* Thresholds** | | |
| --- | --- | --- | --- | --- | --- | --- | --- | --- | --- |
| **Cancer type** | **BAE** | **MAE** | **TE** | **BAE** | **MAE** | **TE** | **BAE** | **MAE** | **TE** |
| **Bladder** | 13.00% | 1.90% | 8.00% | 12.50% | 2.00% | 9.00% | 16.00% | 2.00% | 11.00% |
| **Breast** | 16.50% | 3.00% | 13.00% | 17.00% | 2.50% | 4.00% | 15.00% | 1.80% | 18.00% |
| **Colorectal** | 20.00% | 1.70% | 15.00% | 20.00% | 1.50% | 5.00% | 11.50% | 1.50% | 14.00% |
| **Esophagus** | 20.00% | 2.50% | 12.00% | 20.00% | 2.50% | 12.00% | 25.00% | 2.30% | 15.00% |
| **Gastric** | 20.00% | 1.70% | 12.00% | 17.00% | 2.00% | 8.00% | N.A.* | N.A.* | N.A.* |
| **Lung** | 12.10% | 2.70% | 6.00% | 12.00% | 2.00% | 6.00% | 19.00% | 2.00% | 6.00% |
| **Pancreatic** | 15.00% | 4.50% | 7.00% | 19.00% | 4.70% | 5.00% | 15.00% | 1.30% | 13.00% |
| **Prostate** | 15.00% | 1.20% | 10.00% | 19.00% | 2.50% | 4.00% | 17.00% | 3.10% | 9.00% |
| **Skin** | 13.50% | 2.20% | 7.00% | 13.00% | 2.00% | 5.00% | 23.00% | 1.70% | 15.00% |
| **Thyroid** | 22.00% | 3.10% | 11.00% | 11.00% | 0.70% | 10.00% | 20.00% | 1.80% | 9.00% |

* Not available, because in gastric cancer, BAE, MAE and TE are higher in benign than malignant samples.

**Table S16. Computed Sensitivities and Specificities of the Diagnostic Models for the Ten Cancers.**

|  | ***GNAS*** | | ***GRB10*** | | ***SNRPN*** | | **Combined** | |
| --- | --- | --- | --- | --- | --- | --- | --- | --- |
| **Cancer type** | **Sensitivity** | **Specificity** | **Sensitivity** | **Specificity** | **Sensitivity** | **Specificity** | **Sensitivity** | **Specificity** |
| **Bladder** | 94% | 94% | 90% | 82% | 96% | 91% | 99% | 97% |
| **Breast** | 85% | 77% | 94% | 90% | 90% | 87% | 97% | 97% |
| **Colorectal** | 88% | 90% | 96% | 90% | 90% | 81% | 98% | 95% |
| **Esophagus** | 94% | 83% | 84% | 83% | 84% | 87% | 96% | 91% |
| **Gastric** | 96% | 70% | 98% | 78% | N.A.* | N.A.* | 94% | 96% |
| **Lung** | 75% | 74% | 89% | 65% | 73% | 84% | 92% | 87% |
| **Pancreatic** | 94% | 77% | 76% | 77% | 85% | 88% | 94% | 92% |
| **Prostate** | 89% | 76% | 78% | 82% | 91% | 82% | 93% | 88% |
| **Skin** | 81% | 78% | 88% | 89% | 85% | 83% | 98% | 94% |
| **Thyroid** | 88% | 46% | 63% | 92% | 91% | 81% | 93% | 85% |

* Not available, because in gastric cancer, BAE, MAE and TE are higher in benign than malignant samples.
